# An Integrated Metagenomics/Metaproteomics Investigation of the Microbial Communities and Enzymes in Solid-state Fermentation of Pu-erh tea

**DOI:** 10.1038/srep10117

**Published:** 2015-05-14

**Authors:** Ming Zhao, Dong-lian Zhang, Xiao-qin Su, Shuang-mei Duan, Jin-qiong Wan, Wen-xia Yuan, Ben-ying Liu, Yan Ma, Ying-hong Pan

**Affiliations:** 1 College of Longrun Pu-erh Tea, Yunnan Agricultural University, Kunming 650201, Yunnan, China; 2 Institute of Crop Sciences, Chinese Academy of Agricultural Sciences, Beijing 100081, China; 3 Yunnan Research Center on Good Agricultural Practice for Dominant Chinese Medicinal Materials, Yunnan Agricultural University, Kunming 650201, Yunnan, China; 4 Tea Research Institute of Yunnan Academy of Agricultural Science, Menghai 666201, China

## Abstract

Microbial enzymes during solid-state fermentation (SSF), which play important roles in the food, chemical, pharmaceutical and environmental fields, remain relatively unknown. In this work, the microbial communities and enzymes in SSF of Pu-erh tea, a well-known traditional Chinese tea, were investigated by integrated metagenomics/metaproteomics approach. The dominant bacteria and fungi were identified as Proteobacteria (48.42%) and *Aspergillus* (94.98%), through pyrosequencing-based analyses of the bacterial 16S and fungal 18S rRNA genes, respectively. In total, 335 proteins with at least two unique peptides were identified and classified into 28 Biological Processes and 35 Molecular Function categories using a metaproteomics analysis. The integration of metagenomics and metaproteomics data demonstrated that *Aspergillus* was dominant fungus and major host of identified proteins (50.45%). Enzymes involved in the degradation of the plant cell wall were identified and associated with the soft-rotting of tea leaves. Peroxiredoxins, catalase and peroxidases were associated with the oxidation of catechins. In conclusion, this work greatly advances our understanding of the SSF of Pu-erh tea and provides a powerful tool for studying SSF mechanisms, especially in relation to the microbial communities present.

Solid-state fermentation (SSF) is defined as a fermentation process in which microorganisms grow on solid materials without the presence, or in the near-absence, of free liquid^1^. It is a centuries-old microbial technique that has been widely used in the production of traditional foods and alcoholic beverages worldwide^2^,^3^. SSF has gained attention in recent years due to the lower energy requirements, wastewater production and risk of bacterial contamination that accompany the higher product yields^1^,^4^,^5^. At present, SSF products include not only traditional foods, such as vinegar, soy sauce and flavor spices, but also microbial products, such as single-cell protein, spirulina and edible fungi, microbial enzymes, such as amylase, glucosidase, cellulose and pectinase, organic acids, such as citric acid and lactic acid, microbial secondary metabolites, such as gibberellic acid, ergot alkaloids, penicillin and cyclosporin, and other microbial metabolites, such as nucleotides, lipids, vitamins and amino acids^1^,^4^,^6^,^7^,^8^,^9^,^10^.

In the SSF process, microorganisms are the most important participant. It can be placed in natural (indigenous) SSF or pure culture SSF based on the type of microorganism involved. Natural SSF is carried out by mixed cultures in which several microorganisms show symbiotic cooperation^11^,^12^. Thus, an in-depth knowledge of the microorganisms is essential to understand the mechanism of SSF, especial natural SSF.

Culture-independent molecular techniques, such as denaturing gradient gel electrophoresis (DGGE), temporal temperature gradient gel electrophoresis (TTGE), single stranded con formation polymorphism (SSCP), real-time quantitative PCR (qPCR), the construction and analysis of 16S rRNA gene libraries, terminal restriction fragment length polymorphism (TRFLP) and next generation sequencing (NGS) techniques, have been widely used to analyze the microbiota of food fermentation, including the SSF process, increasing our knowledge of microbial diversity, population structure and dynamics^13^,^14^,^15^. These studies were based on the analysis of 16S rRNA gene sequences, which provides useful information on microbial composition; however, the microbial enzymes still remain unknown.

Metaproteomics, which is the identification of all the proteins expressed at a given time within an ecosystem, and has been applied in diverse environments, such as soil, sediments, marine, freshwater, wastewater, human intestinal tract, human oral cavity and animal guts, as well as natural and bioengineered systems^16^,^17^,^18^,^19^,^20^. However, studying the microbial enzymes in the SSF process using the metaproteomics approach has still been limited.

Post-fermented Pu-erh tea (pu-erh shucha, PFPT), a well-known traditional Chinese tea, is produced by a natural SSF process using sun-dried green tea leaves (*Camellia sinensis* var. *assamica* (JW Masters) Kitamura) as the raw material^21^. The microbial community and its enzymes in SSF is thought to be important for the tea to develop its characteristic properties, including a reddish-brownish color, mellow taste, stale flavor, and long-term storage, as well as the health benefits of Post-fermented Pu-erh tea, which include hypolipidemic, antimutagenic, antioxidative, antitumor, antiobesity and toxicity suppressing activities^22^,^23^. Microorganisms involved in the SSF of Pu-erh tea have been mainly studied using culture-based approaches^24^,^25^,^26^,^27^,^28^,^29^,^30^, and recently several culture-independent approaches^31^,^32^,^33^. However, as far as we know, there are little reports on the microbial enzymes during the SSF of Pu-erh tea.

In this work, the microbial communities and enzymes in a SSF of Pu-erh tea were investigated using 454 pyrosequencing and LC-MS/MS approaches, respectively. This study improves our knowledge regarding the formation of the characteristic properties and health benefits of Post-fermented Pu-erh tea, and studies the mechanisms of SSF using the metagenomics/metaproteomics approach.

## Results and Discussion

**Figure 1:**
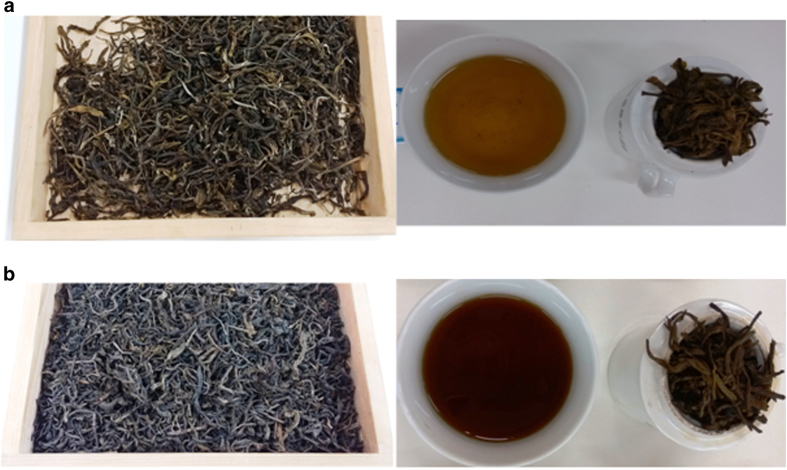
Sensory evaluation of tea leaves collected on day 0 (**a**) and 21 (**b**) during the SSF of pu-erh tea. The sensory evaluation was developed according the Chinese standard (GB/T 23776-2009) as follow: 3 g of dried tea leaves were infused in 150 ml boiled water for 5 min; the tea leaves, the tea liquor and leaves after infused were showed, respectively.

To better understand the SSF of traditional Chinese Pu-erh tea, triplicate laboratory fermentation was performed. The sample collected on day 21 was selected for further metagenomics and metaproteomics analyses. The comparison of raw material (day 0) and fermented tea leaves collected on day 21 are presented in Fig. 1 and a comparison of the isolated chemical compounds are presented in Table 1. After SSF, the tea leaves became dark, and after a water infusion, the liquid became reddish-brownish and the fermented tea leaves softened. The contents of tea polyphenols, free amino acids, EGC, C, EC, EGCG, GG, ECG and TR decreased significantly (P < 0.05); however, the contents of GA and TB increased significantly (P < 0.05). The content of CAF was slight increased (P > 0.05). This change in the chemical compounds during SSF of Pu-erh tea was in accord with previous reports^34^.

After removing low quality and chimeric sequences, 5,489 and 5,323 sequences containing 2,319 and 39 phylogenetic OTUs, respectively, were obtained from the bacterial and fungal PCR amplicons, respectively (Table 2). Rarefaction curves with a 3% cutoff are shown in Figure S1. Additionally, the Chao1 estimation, and ACE and Shannon indices are shown in Table 2. Using Ribosomal Database Project (RDP) identifiers, the 16S rRNA genes were classified into 16 phyla, including Proteobacteria (48.42%), Firmicutes (19.91%), Actinobacteria (16.91%), Cyanobacteria (9.95%) and Bacteroidetes (3.79%). Based on the pyrosequencing analysis of the ITS region of the 18S rRNA gene, the dominant fungi were classed into genus *Aspergillus* accounting for 94.98% of the total sequences (Table 2). This corroborated previous reports that the major fungi involved in the SSF of Pu-erh tea were *A. niger* and *B. adeninivorans*^32^, and *A. niger*, *S. cerevisiae* and *P. glabrum*^35^. The results of the community structure analysis indicated that there is a high diversity level of bacteria, which was corroborated by the bacterial diversity indices being significantly greater than those of fungi (Table 2). Additionally, the bacterial rarefaction curve did not approach a plateau at a similar level (Figure S1). Fungi belonging to *Aspergillus* were identified as dominant throughout the SSF process of PFPT,

It was hypothesized that microbial extracellular enzymes play key roles in the transformation of the chemical constituents during SSF of Pu-erh tea^22^, thus we aimed to investigate the microbial secreted proteins but not whole microbial cellar proteins or proteins of tea leaves. To extract the microbial secreted proteins, we first measured the amounts of protein suspended in extraction buffer B at various time (2, 4, 6, 8 and 10 min) (Figure S2). A one-way ANOVA analysis showed that there were no significant difference in the amounts of proteins extracted among the various times (P > 0.05). However, the supernatants became darker as the time increased. Thus, fermented tea leaves were suspended in the extraction buffer at 2 min during further experiments.

**Figure 2:**
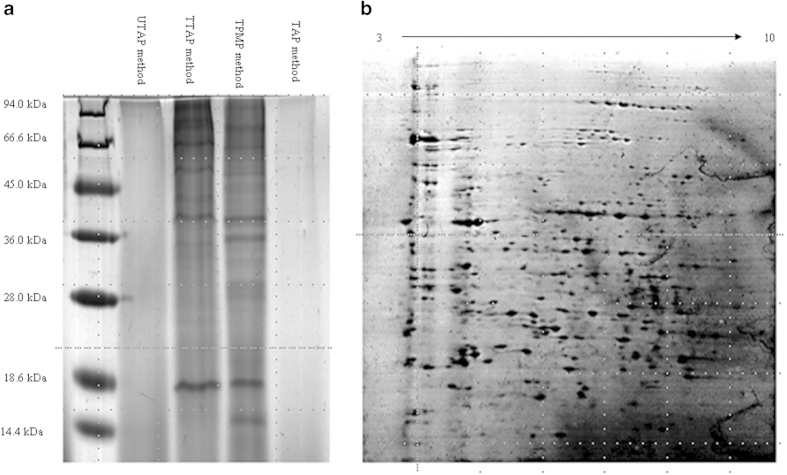
Proteins separated by SDS-PAGE after extraction from fermented tea leaves using various methods (**a**) Proteins separated by 2-DE after extraction from fermented tea leaves using the Tris-HCl/phenol and methanol precipitation (TPMP) method (**b**) TAP method indicates proteins extracted using TCA/acetone precipitation; TTAP method indicates proteins extracted using Tris-HCl and TCA/acetone precipitation; and UTAP method indicated protein extracted using urea/thiourea and TCA/acetone precipitation.

Due to the complexity of the SSF of tea leaves, preparing high-quality protein samples is crucial for the metaproteomics analysis. Proteins were extracted from fermented tea leaf samples using four extraction methods. SDS-PAGE showed that more and clearer bands with lighter background were obtained using the TPMP method (Fig. 2a). Proteins were prepared using this method and subjected to 2-DE analysis (Fig. 2b). More than 200 reproducible protein spots were obtained and yielded acceptable profiles of proteins in 2-D gels. Thus, we developed a protocol for the metaproteomic analysis of microbial proteins in microbial fermented tea leaves characterized by a high polyphenol content. This protocol is based on the phenol extraction method. Phenol extraction is widely used in protein extraction from various matrices, such as plant, sediment and soil^36^, and has been used in the proteomic analysis of plant extracts containing polyphenols^37^. In this work, we showed that microbial proteins could be extracted and purified from fermented tea leaves using Tris-HCl/phenol extractions followed by ammonium acetate–methanol precipitation.

**Figure 3:**
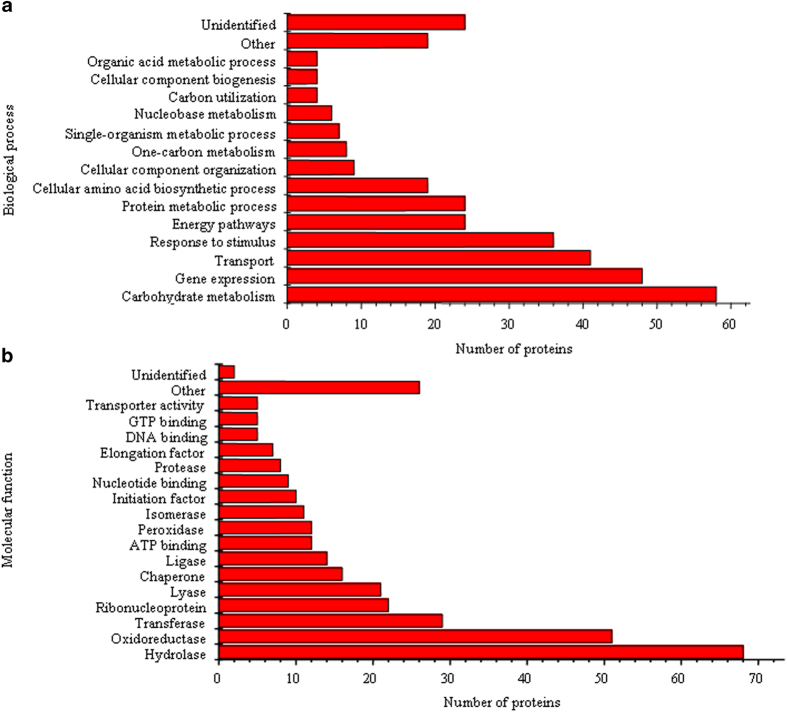
Biological process (**a**) and Molecular function (**b**) categories of identified proteins based on a gene ontology analysis. The categories including more than 1% of the total identified proteins are presented.

The proteins in fermented tea leaves collected on day 21 were prepared and subjected to an LC-MS/MS analysis (Figure S3). In total, 335 proteins, such as inorganic pyrophosphatase, a 78-kDa glucose-regulated protein homolog, alcohol dehydrogenase 1 and catalase-peroxidase (Table S1), with at least two unique peptides were identified when searched against the NCBI nr (non-redundant protein sequence) bacterial and fungal database. According to GO annotations, 311 identified proteins were classified into 28 Biological Process (BP) categories (Fig. 3a), and 333 identified proteins were classified into 35 Molecular Function (MF) categories (Fig. 3b). Highly represented categories were associated with carbohydrate metabolism (18.65% of the GO annotated proteins), gene expression (15.45%), transport (13.18%) and response to stimulus (11.58%) in BP, and hydrolase (20.42%), oxidoreductase (15.32%) and transferase (8.71%) in MF.

The taxonomic distribution of the identified proteins based on the non-redundant protein groups suggested that 40 of the identified proteins were associated with bacteria spanning five phyla and 24 genera. Most bacterial proteins were associated with Proteobacteria (75%) hosts. The metagenomics survey also demonstrated that the dominant bacteria were Proteobacteria (48.42%). The relative percentages of bacterial phyla assigned based on the 16S rRNA gene sequences correlated well with those assigned through proteomics (P < 0.05) and a comparison of the bacterial taxonomic groups at the phylum level is presented in Figure S4. In total, 295 identified proteins were associated with fungi spanning four phyla and no_rank_fungi. Most fungal proteins belonged to Ascomycota (96.6%) hosts mostly from the genus *Aspergillus* (58.68%). The relative percentages of fungal phyla and genera assigned based on the 18S rRNA gene sequences correlated well with those assigned through proteomics (P < 0.01), and a comparison of the fungal taxonomic groups at the genus level is presented in Figure S5.

The metagenomics analysis showed that the bacterial communities of fermented tea leaves have a high diversity level; however, 88.06% of the identified proteins were assigned to fungi by the proteomic analysis. The metagenomics analysis revealed that *Aspergillus* was the dominant fungal genus (94.98%) and metaproteomics demonstrated that *Aspergillus* was also the major source of fungal proteins (58.68%) and all identified proteins (50.45%). Thus, direct evidence between the microorganisms and enzyme producers was provided by this integrated study, which demonstrated that fungi, especially fungi belonging to the genus *Aspergillus*, play important roles during the SSF of Pu-erh tea.

Though microbial extracellular enzymes was hypothesized to responsible for the characteristics of Post-fermented Pu-erh tea^23^, which extracellular enzymes were present during the SSF of Pu-erh tea leaves had not been reported. In this work, 42 identified proteins were classified as secreted proteins or located in extracellular regions, such as alkaline protease, endo-1,4-β-xylanase, pectate lyase and pectinesterase (Table S2). According to the GO annotations, α-L-arabinofuranosidase, endo-1,4-β-xylanase, exo-1,4-β-xylosidase, α-glucuronidase, and α-*N*-arabinofuranosidase were involved in the hydrolysis of xylan, while pectate lyase, pectin lyase, rhamnogalacturonate lyase, endo-xylogalacturonan hydrolase, arabinan endo-1,5-α-L-arabinosidase and arabinogalactan endo-1,4-β-galactosidase were involved in the degradation of pectin. Additionally, β-glucosidase and 1,4-β-D-glucan cellobiohydrolase were involved in the degradation of cellulosic biomass and α-*N*-arabinofuranosidase was involved in the degradation of arabinoxylan. Xylan, pectin, cellulose and arabinoxylan are polysaccharides of the plant cell wall. Thus, our metaproteomics analysis demonstrated that microbial extracellular enzymes could degrade the tea plant cell wall, leading to the maceration and soft-rotting of tea leaves. This was supported by the observation of soft-rotting tea leaves during the SSF of Pu-erh tea and by Wang *et al.*, who showed that the surfaces of tea leaves were covered by microorganisms and the cells structures were largely disrupted after SSF^38^. Additionally, the degradation of polysaccharides and the hydrolysis of pectin may be associated with the mellow taste of Pu-erh tea.

HPLC and spectraphotometric method showed that the polyphenol, catechin, and TR contents were decreased significantly (P < 0.05); however, the TB content was increased significantly (P < 0.05). This may be due to the oxidization of catechins. During the fermentation of black tea, the oxidization of catechins were catalyzed by endogenous polyphenol oxidase (PPO) and peroxidase (POD). The oxidation products, such as theaflavins and thearubigins, contribute to the color and the taste of black tea^39^,^40^. In this work, PPO was not identified; three catalases, a catalase-peroxidase and two peroxiredoxins were identified. We suggested these enzymes, especial catalase (Q877A8), catalase-peroxidase (A2Q7T1) and peroxiredoxin (Q5ASN8), which are secreted enzymes, may catalyze the oxidization of catechins during the SSF of Pu-erh tea.

## Conclusion

The microbial communities and enzymes in SSF of Pu-erh tea leaves were investigated by an integrated metagenomics/metaproteomics approach. Through this integrated analysis, we know which microorganisms are present, as well as which proteins are produced, and by which microorganism during this SSF of Pu-erh tea. Some enzymes that are associated with the formation of the characteristic properties of Post-fermented Pu-erh tea were identified. This novel information improves our knowledge of the SSF of Pu-erh tea.

## Methods

### Pu-erh tea fermentation, sample collection and chemical compounds analysis

Sun-dried green tea, used as the raw material for the fermentation of Pu-erh tea, was purchased from Puer City, Yunnan Province, China. A 30 kg sample of the green tea leaves was mixed with 15 L of tap water to give a solid content of ~65% (w/v). During fermentation, the leaves were mixed to ensure homogeneity and tap water was added to keep the solids constant at 65–75% (as judged by the manufacturer). Triplicate fermentations were performed. Samples were collected from the tank every 7 days and subjected to sensory evaluation as described by GB/T 23776-2009^41^. The fermentation process was stopped when the fermented tea mass was reddish-brown and free from the astringent taste (~35 days). The sample collected on day 21 was stored at −80 °C and selected for further metagenomics and metaproteomics analyses.

The contents of polyphenols and free amino acids in the tea leaves were determined using the spectraphotometric method based on FeSO_4_ and the ninhydrin assay described by Liang *et al.,* respectively^42^. The main tea pigments including theabrownin (TB), theaflavin (TF) and thearubigin (TR) were analyzed using the spectrophotometry method described by Wang *et al.*^38^. The composition of gallic acid (GA) and caffeine (CAF) , as well as the catechins, including (+)- catechin (C), (−)-epicatechin (EC), (−)-epigallocatechin (EGC), (−)-epicatechin 3-*O*-gallate (ECG), (−)-epigallocatechin 3-*O*-gallate (EGCG), 1,4,6-tri-*O-*galloyl-*β*-D-glucose(GG) level in the tea leaves was determined by high-performance liquid chromatography (HPLC) using an Agilent 1200 series system and a TSK-GEL ODS-80TM column (4.6 mm i.d. × 250 mm, Tosoh, Japan). The detailed approaches are described in Supplementary material 1.

### Metagenomics analysis

Universal primers 27F (5′-AGAGTTTGATCCTGGCTCAG-3′) and 533R 5′-TTACCGCGGCTGCTGGCAC-3′), ITS1 (5′-TCCGTAGGTGAACCTGCGG-3′) and ITS4 (5′-TCCTCCGCTTATTGATATGC-3′), incorporating the FLX Titanium adapters, targeting the V1–V3 region of bacterial 16S rRNA and the ITS of fungal 18S rRNA, respectively, were chosen for the amplification and subsequent pyrosequencing of the PCR products. DNA extraction, PCR amplification, amplicon quantitation and pyrosequencing were performed at Majorbio Bio-Pharm Technology Co., Ltd., Shanghai, China. Detail approaches are provided in Supplementary material 1.

Data preprocessing was performed mainly using MOTHUR software^43^,^44^. Chimeric sequences were excluded using the chimera.uchime command with default parameters^45^. Sequences with similarities > 97% were clustered into one operational taxonomic unit (OTU) using MOTHUR. The taxonomical assignment of each OTU was performed using the classify.seqs command (Naïve Bayesian Classifier) against the SILVA 16S rRNA gene database (release 111) at an 80% confidence level^46^,^47^,^48^. Community richness and diversity indices (Chao1 estimator, abundance-based coverage estimator (ACE) and Shannon indices, respectively) and rarefaction curves were obtained using MOTHUR^49^.

The 454 pyrosequencing data generated for this study were submitted to the Sequence Read Archive (SRA) and are available under project SRR1596332

### Method for extracting microbial proteins from fermented tea leaves

**Figure 4:**
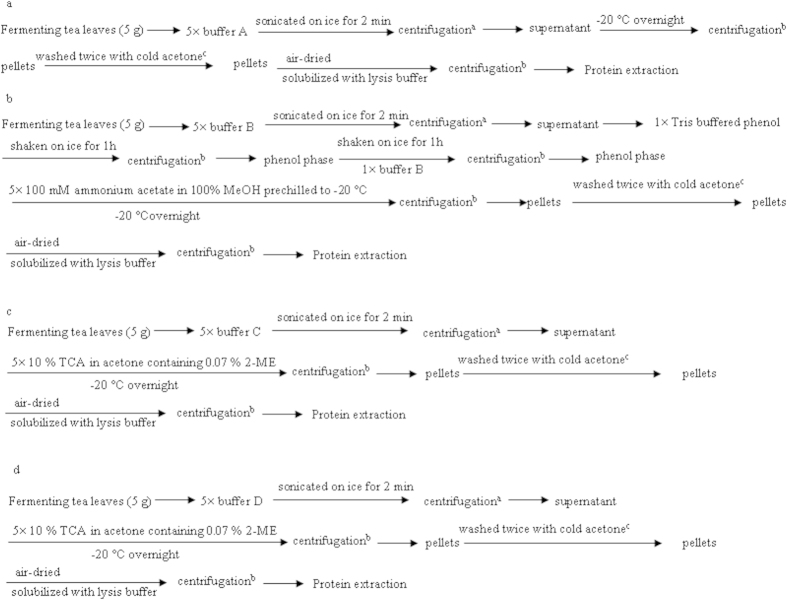
Summary of protein extractions from fermented tea leaves by trichloroacetic acid (TCA)/acetone precipitation (**a**), Tris-HCl/phenol and methanol precipitation (**b**), Tris-HCl and TCA/acetone precipitation (**c**) and urea/thiourea and TCA/acetone precipitation (**d**). ^a^Centrifugation (4,000 × g at 4 °C for 5 min); ^b^centrifugation (12000 × g at 4 °C for 15 min); ^c^pellets were suspended in cold acetone and kept at −20 °C for 1 h followed with centrifugation at 12,000 × g at 4 °C for 20 min.

To extract microbial proteins that met the requirements for a metaproteomic analysis from fermenting tea leaves characterized by a high content of polyphenols, four modified plant proteome sample preparation methods were used and repeated three times. A summary of these procedures is supplied in Fig. 4 and detailed procedures are described as follows:

#### (1) Protein extraction by trichloroacetic acid (TCA)/acetone precipitation (TAP method)

Five grams of tea leaves were suspended in extraction buffer A [10% TCA in acetone, 0.07% 2-mercaptoethanol (2-ME) and 2% polyvinylpolypyrrolidone (PVPP)] and then sonicated on ice for 2 min. Then, the samples were centrifuged at 4,000 × *g* at 4 °C for 5 min, and the supernatants were collected and kept at −20 °C overnight and the tea leaves were discarded. The supernatant was then centrifuged at 12,000 × *g* at 4 °C for 20 min. The pellets were washed twice with cold acetone as follows: pellets were suspended in cold acetone and kept at −20 °C for 1 h followed by centrifugation at 12,000 × *g* at 4 °C for 20 min. The remaining pellets were air-dried, solubilized with lysis buffer [7 M urea, 2 M thiourea, 4% CHAPS, 40 mM Tris-Base, 40 mM dithiothreitol (DTT) and 2% Pharmalyte, pH 3–10], incubated at room temperature for 1 h and then centrifuged at 12,000 × *g* at 4 °C for 15 min. The supernatants were collected in 1.5 mL tubes and stored at −80 °C for later use (Fig. 4a).

#### (2) Protein extraction by Tris-HCl/Phenol and Methanol precipitation (TPMP method)

Five grams of tea leaves were suspended in extraction buffer B (50 mM Tris-HCl buffer pH 7.5, 100 mM KCl, 50 mM EDTA, 5 mM DTT, 2% PVPP and 30% sucrose) and sonicated on ice for 2 min. Then, the samples were centrifuged at 4,000 × *g* at 4 °C for 5 min, the supernatants were collected and the tea leaves were discarded. Tris buffered phenol was added to the samples in a 1:1 ratio, samples were then shaken on ice for 1 h, centrifuged at 12000 × *g* for 15 min at 4 °C. The upper phenol phase was transferred into a new 1.5 mL tube and extracted with an equal volume of fresh extraction buffer B. Extracted proteins were precipitated from the phenol phase by adding five volumes of 100 mM ammonium acetate in 100% MeOH prechilled to −20 °C, incubated overnight at −20 °C, and then collected by centrifugation at 12000 × *g* for 15 min at 4 °C. The protein pellets were washed twice with cold acetone. The remaining pellets were air-dried and solubilized with lysis buffer. The supernatants were collected in 1.5 mL tubes and stored at −80 °C for later use (Fig. 4b).

#### (3) Protein extraction by Tris-HCl and TCA/acetone precipitation (TTAP method)

Five grams of tea leaves were suspended in extraction buffer C (65 mM Tris HCl buffer pH 6.8, 0.5% SDS, 10% glycerin, 5% 2-ME and 2% PVPP) and sonicated on ice for 2 min. Then, the supernatants were collected by centrifugation at 4,000 × *g* at 4 °C for 5 min. Extracted proteins were precipitated from the supernatant by adding five volumes of 10% TCA in acetone containing 0.07% 2-ME prechilled to −20 °C, incubated overnight at −20 °C, and then collected by centrifugation at 12000 × *g* for 15 min at 4 °C. The protein pellets were washed twice with cold acetone, and the remaining pellets were air-dried and solubilized with lysis buffer. The supernatants were collected in 1.5 mL tubes and stored at −80 °C for later use (Fig. 4c).

#### (4) Protein extraction by urea/thiourea and TCA/acetone precipitation (UTAP method)

Five grams of tea leaves were suspended in extraction buffer D (5 M urea, 2 M thiourea, 2% SDS, 2% Triton-114, 5 mM DTT and 2% PVPP) and sonicated on ice for 2 min. Then, the supernatants were collected through a centrifugation at 4,000 × *g* at 4 °C for 5 min. Extracted proteins were precipitated from the supernatant by adding five volumes of 10% TCA in acetone containing 0.07% 2-ME prechilled to −20 °C, incubated overnight at −20 °C, and then collected by centrifugation at 12000 × *g* for 15 min at 4 °C. The protein pellets were washed twice with cold acetone, and the remaining pellets were air-dried and solubilized with lysis buffer. The supernatants were collected in 1.5 ml tubes and stored at −80 °C for later use (Fig. 4d).

#### Validating the quality of protein extractions

The protein concentration was determined by the Bradford method using bovine serum albumin (BSA) as a standard. SDS-polyacrylamide gel electrophoresis (SDS-PAGE) and two-dimensional gel electrophoresis (2-DE) were used to validate the quality of the protein extractions. SDS-PAGE was performed using a 5% stacking gel and 12.5% separating gel in the Mini-p4 System (BioRad, California, USA). The first-dimension isoelectric focusing (IEF) was performed using the EttanIII system (GE Healthcare, NJ, USA), and the 2-DE was performed using the Ettan Daltsix electrophoresis system (GE Healthcare, NJ, USA) according to the manufacturer’s instructions. Detailed approaches are provided in Supplementary information 1. After electrophoresis, the gel was visualized with Colloidal Coomassie Brilliant Blue G-250 using the Blue silver method^50^ and imaged.

#### LC-MS/MS analysis and data processing

Microbial proteins were prepared from tea leaves fermented for 21 days using the Tris-HCl/phenol and methanol precipitation (TPMP) method. For each fermented sample, three independent extractions were performed. Then 9 samples of proteins were pooled and purified using 2-D Clean-up kit (GE Healthcare, NJ, USA); 1.5 mg of proteins was digested with trypsin according to the Filter Aided Sample Preparation (FASP) protocol^51^ for each sample replicate. LC-MS/MS analyses of the peptide extracts were performed using an Easy-nLC1000 coupled to a Q-Exactive mass spectrometer (Thermo Fisher Scientific, Bremen, Germany) for each sample replicate. Lyophilized peptides were reconstituted in 14 μL of 2% acetonitrile and 0.05% trifluoroacetic acid, and an aliquot of 5 μL (approximatively 7 μg total peptide) was loaded onto a C18 analytical column (2 μm, 100 Å, 75 μm ID × 15 cm) (Thermo Fisher Scientific, Bremen, Germany). LC solvents included solvent A, water containing 0.1% formic acid, and 3% of solvent B, acetonitrile containing 0.1% formic acid. The LC gradient was set as follows: 0 min, 3% B; 10 min, 8% B; 88 min, 20% B; 103 min, 30% B; 113 min, 90% B; 113–120 min, solvent B was kept at 90%; and the flow rate was 350 nL/min. During the entire chromatographic process, the linear trap quadrupole (LTQ) mass spectrometer was operated in a data-dependent MS/MS mode with the following parameters: nanospray voltage (2.4 kV), heated capillary temp 250 °C, full scan *m*/*z* range (300–1800), resolutions (70,000 at m/z200). The 15 most intense precursors were selected for higher-energy collisional dissociation (HCD) fragmentation with a normalized collision energy of 27%. HCD spectra were acquired in the Orbitrap with a 17,500 at m/z 200 resolution and a starting mass of m/z 100.

Raw data were processed for database searching using Thermo Proteome Discoverer software (v1.0 build 43, Thermo Fisher Scientific) and were used to run MASCOT searches and Mascot (Matrix Science, London, UK) at the in-house server to perform database comparisons against all bacterial and fungal entries in the National Center for Biotechnology Information database^52^. Only when two or more peptide fragments were matched to the same environmental protein was the protein (and its host cell) considered “identified.” The highest score for a given peptide mass (best match to that predicted in the database) was used to identify parent proteins. Gene Ontology (GO) annotations for the identified proteins were assigned according to those reported in the uniprot database ( http://www.uniprot.org). The Gramene Ontology Search ( http://archive.gramene.org) was used for plotting GO annotation results at GO level 2.

## Author Contributions

M.Z., Y.M. and Y.P. planned the research project, analyzed the data and wrote the manuscript; D.Z. and X.S. did most of the experiments; S.D. and J.W. did chemical compound analysis; W.Y and B.L. did the fermentation; and all authors reviewed the manuscript.

## Additional Information

**How to cite this article**: Zhao, M. *et al.* An Integrated Metagenomics/Metaproteomics Investigation of the Microbial Communities and Enzymes in Solid-state Fermentation of Pu-erh tea. *Sci. Rep.* **5**, 10117; doi: 10.1038/srep10117 (2015).

## Supplementary Material

Supplementary Information

## Figures and Tables

**Table 1:** A comparison of the chemical compounds in raw materials (day 0) and fermented tea leaves collected on day 21.

**Sample**	**Raw material**	**fermenting tea leaves collected on day 21**
tea polyphenols (%)	37.86 ± 1.72	15.71 ± 0.67^*^
free amino acid (%)	2.52 ± 0.02	1.01 ± 0.09^*^
EGC (mg/g)	29.6 ± 7.94	15.72 ± 5.08^*^
C (mg/g)	9.83 ± 0.76	2.90 ± 0.68^*^
EC (mg/g)	13.70 ± 0.12	11.07 ± 1.97^*^
EGCG (mg/g)	59.72 ± 1.79	2.64 ± 0.87^*^
GG (mg/g)	4.19 ± 0.22	0.65 ± 0.18^*^
ECG (mg/g)	39.63 ± 1.35	1.78 ± 0.83^*^
GA (mg/g)	3.88 ± 0.18	7.79 ± 1.16^*^
CAF (mg/g)	28.50 ± 0.19	29.20 ± 3.34
TF (%)	0.35 ± 0.01	0.34 ± 0.08
TR (%)	7.01 ± 0.08	5.97 ± 0.08^*^
TB (%)	3.23 ± 0.08	11.32 ± 0.87^*^

Note: *indicated there is significant difference (P < 0.05).

**Table 2:** Operational taxonomic unit (OTU) analyses, abundance estimators [Chao1 and abundance-based coverage estimator (ACE)], diversity estimators (Shannon and Simpson) and major microbes at genus level (>1%) in fermented tea leaves based on the V1–V3 hypervariable regions of the bacterial 16S rRNA gene and the ITS regions of the fungal 18S rRNA gene.

	**Gene**	**No. of high quality reads**	**OTUs**	**Major microbes at genus level (>1%)**	**Coverage (%)**	**Richness estimators**		**Diversity indices**	
						ACE	Chao1	Shannon-Weaver index	Simpson
Bacteria	16S rRNA gene	5489	2319	*Pseudomonas* (17.82%), *Lactococcus* (12.33%), unclassified_Cyanobacteria (9.53%), unclassified_Enterobacteriaceae (7.69%), *Curtobacterium* (7.36%), *Achromobacter* (4.74%), *Acinetobacter* (3.99%), *Rhodococcus* (2.97%), *Stenotrophomonas* (2.56%), *Enterococcus* (2.46%), *Erwinia* (2.17%), *Streptomyces* (2.10%), *Enterobacter* (1.51%), *Paenibacillus* (1.33%), *Flavobacterium* (1.27%), *Bacillus* (1.18%), *Microbacterium* (1.15%), *Klebsiella* (1%), Other16.85%	67.33	18573	8450	6.52	0.01
Fungi	18S rRNA gene	5323	39	*Aspergillus* (94.98%), *Rhizomucor* (4.88%), other (0.14%)	99.64	103	60	0.45	0.84
