# Supplementary material for: An Integrated Metagenomics/Metaproteomics Investigation of the Microbial Communities and Enzymes in Solid-state Fermentation of Pu-erh tea
Source: Sci Rep. 2015 May 14;5:10117. doi: 10.1038/srep10117 (PMC4431464; doi:10.1038/srep10117)
Supplement: Supplementary Information [file srep10117-s1.doc]

**An Integrated Metagenomics/Meta****proteomics Investigation of the Microbial** **Communities and Enzymes in Solid-state Fermentation of Pu-erh tea**

Ming Zhao^1, 3^, Dong-lian Zhang^1^, Xiao-qin Su^1^, Shuang-mei Duan^1^, Jin-qiong Wan^1^, Wen-Xia Yuan^1^, Ben-ying Liu^4#^, Yan Ma^1#^, Ying-Hong Pan^2#^

^1^College of Longrun Pu-erh Tea, Yunnan Agricultural University, Kunming 650201, Yunnan, China,

^2^Institute of Crop Sciences, Chinese Academy of Agricultural Sciences, Beijing 100081, China,

^3^Yunnan Research Center on Good Agricultural Practice for Dominant Chinese Medicinal Materials, Yunnan Agricultural University, Kunming 650201, Yunnan, China,

^4^Tea Research Institute of Yunnan Academy of Agricultural Science, Menghai 666201, China

*Correspondence to [yhpan@caas.net.cn](mailto:yhpan@caas.net.cn); [mayan202@163.com](mailto:mayan202@163.com); [liusuntao@126.com](mailto:liusuntao@126.com)

#equal author contribution.

**Supplementary material 1 Detailed of Materials and methods**

Detailed analysis of tea chemical compounds

The content of the tea polyphenols was determined using the spectraphotometric method based on FeSO_4_ and described by Liang *et al.* ^1^. Free amino acids in fermenting tea leaves were determined using the ninhydrin assay method ^1^*.* The main tea pigments, including theabrownin (TB), theaflavin (TF) and thearubigin (TR), were analyzed using the spectrophotometric method described by Wang *et al.* ^2^. The composition of gallic acid (GA), and caffeine (CAF) , as well as catechins, including (+)-catechin (CA), , (−)-epicatechin (EC), (−)-epigallocatechin (EGC), (−)-epicatechin 3-*O*-gallate (ECG), (−)-epigallocatechin 3-*O*-gallate (EGCG), 1,4,6-tri-*O-*galloyl-*β*-D-glucose (GG)in tea leaves was determined by high-performance liquid chromatography using an Agilent 1200 series HPLC system consisting of an LC-20AB solvent delivery unit, a SIL-20A autosampler, a CTO-20A column oven (40°C), a G1314B VWD (280 nm), and an LC Ver1.23 workstation (Agilent, California, USA ). Separation was completed using the TSK-GEL ODS-80TM column (4.6 mm i.d. × 250 mm, Tosoh, Japan). The mobile phases were eluted in solvent A (0.05 M H_3_PO_4_-H_2_O and 5% CH_3_CN) and solvent B (0.05 M H_3_PO_4_-H_2_O and 80% CH_3_CN). Elution conditions and flow rates were: in 0–25 min solvent A was reduced from 95% to 55% and B from 5% to 45% (linear gradient); at 26 min solvent B increased to 80%; at 26–35 min solvent B increased from 80% to 100%; at 35–39.5 min solvent B was kept at 100%; at 39.5–39.8 min solvent A increased from 0 to 95%; and at 40–45 min solvent A was kept at 95%; the flow rate was 1 mL/min. The temperature of the column oven was maintained at 40°C. The injection volume was 10 μL. The chemical compounds were identified in the tea liquids by comparing the retention times of the peaks with those of the standards. Each tea was extracted twice and each extraction was analyzed twice.

Detailed DNA extractions, PCR amplifications, amplicon quantitation and pyrosequencing protocols

Metagenomic DNA from fermenting tea leaves was extracted using the E.Z.N.A.^TM^ Soil DNA Kit (Omega Bio-Tek, Inc., USA) according to the manufacturer’s instructions. The transGen AP221-02 kit with TransStart Fastpfu DNA polymerase (TransGen Biotech, Beijing, China) was used for PCR. Each PCR consisted of 0.4 μL Fastpfu DNA Polymerase, 4 μL 5-fold FastPfu Buffer, 2 μL 2.5 mM dNTPs, 0.8 μL of each primer, 10 ng of DNA and ddH_2_O to a final concentration of 20 μL.

To analyze the taxonomic composition of the bacterial community, universal primers 27F (5′-AGAGTTTGATCCTGGCTCAG-3′) and 533R (5′-TTACCGCGGCTGCTGGCAC-3′), incorporating the FLX Titanium adapters targeting the V1–V3 region of 16S rRNA gene, were chosen for the amplification and subsequent pyrosequencing of the PCR products. The amplification program consisted of an initial denaturation step at 95°C for 2 min. This was followed by 25 cycles, where 1 cycle consisted of 95°C for 30 s (denaturation), 55°C for 30 s (annealing) and 72°C for 30 s (extension), followed by a final extension of 72°C for 5 min. To analyze the taxonomic composition of the fungal community, universal primers ITS1 (5′-TCCGTAGGTGAACCTGCGG-3′) and ITS4 (5′-TCCTCCGCTTATTGATATGC-3′), incorporating the FLX Titanium adapters targeting the 18S rRNA gene, were chosen for the amplification and subsequent pyrosequencing of the PCR products. The amplification program consisted of an initial denaturation step at 95°C for 2 min. This was followed by 30 cycles, where 1 cycle consisted of 95°C for 30 s (denaturation), 55°C for 30 s (annealing) and 72°C for 30 s (extension), followed by a final extension of 72°C for 5 min. All PCR products were visualized on agarose gels (2% in TBE buffer) containing ethidium bromide. For each sample, three independent PCRs were performed. The triplicate products were pooled and purified using AxyPrep PCR Clean-up Kit (Axygen Biosciences, CA, USA). The DNA concentration of each PCR product was determined using a Quant-iT PicoGreen doublestranded DNA assay (Invitrogen, Germany), and the quality was analyzed using an Agilent 2100 bioanalyzer (Agilent, USA). Amplicon pyrosequencing was performed from the A-end using a 454/Roche A sequencing primer kit on a Roche Genome Sequencer GS-FLX Titanium platform at Majorbio Bio-Pharm Technology Co., Ltd., Shanghai, China.

Detailed approaches to validate the quality of protein extractions

The protein concentrations were determined using the Bradford method with bovine serum albumin (BSA) as the standard ^3^. SDS-polyacrylamide gel electrophoresis (SDS-PAGE) and two-dimensional gel electrophoresis (2-DE) were used to validate the quality of the protein extractions. SDS-PAGE was performed in 5% stacking gels and 12.5% separating gel using the Mini-p4 System (BioRad, California, USA ). The first-dimension isoelectric focusing (IEF) was performed using 13 cm nonlinear IPG strips (pH 3–10) in the EttanIII system (GE Healthcare, NJ, USA), according to the manufacturer’s instructions. IPG strips were actively rehydrated (16 h at 50 V) with 250 μL of rehydration buffer (8 M urea, 2% CHAPS, 0.5% ampholytes and 0.002% bromophenol blue) containing 120 μg of protein. Voltage settings for IEF were 100 V for 2 h, 200 V for 2 h, 500 V for 2 h, 1000 V for 2 h, followed by 1000–5000 V (linear gradient) over 1 h and 5000–9000 V (linear gradient) over 3 h, and finally by running at 9000 V to achieve 7 kV/h. After IEF, strips were incubated with equilibration buffer 1 [50 mM Tris-HCl (pH 8.8), 6 M urea, 30% glycerol, 2% SDS, 0.002% bromophenol blue and 1% DTT] and equilibration buffer 2 [50 mM Tris-HCl (pH 8.8), 6 M urea, 30% glycerol, 2% SDS, 0.002% bromophenol blue and 2.5% iodoacetamide] for 20 min each and subsequently placed onto a 12.5% polyacrylamide gel (26 × 32 cm) using a Tris-glycine buffer system. Strips were overlaid with an agarose sealing solution (0.25 M Tris base, 1.92 M glycine, 1% SDS, 0.5% agarose and 0.002% bromophenol blue) using an Ettan Daltsix electrophoresis system (GE Healthcare, NJ, USA). The initial 2-D eletrophoresis setting was 1 W per gel for 1 h, followed by a separation run using 5 W per gel until the bromophenol blue front reached the bottom of the gels. After electrophoresis, the gel was visualized with Colloidal Coomassie Brilliant Blue G-250 using the Blue silver method ^4^ and imaged.

Table S1 List of the identified proteins in Pu-erh tea

| Accession | Description | Biological process | Molecular function | Subcellular location | Organism | Coverage | Unique Peptides |
| --- | --- | --- | --- | --- | --- | --- | --- |
| Q4WDE1 | 1,2-dihydroxy-3-keto-5-methylthiopentene dioxygenase OS | [Amino-acid biosynthesis](http://www.uniprot.org/keywords/KW-0028) | Dioxygenase, Oxidoreductase | Cytoplasm, Nucleus | *Aspergillus fumigata* | 14.20 | 2 |
| B0XT72 | 1,3-beta-glucanosyltransferase gel1 OS | carbohydrate metabolic process | transferase | Cell membrane | *Aspergillus fumigata* | 5.09 | 2 |
| Q99002 | 14-3-3 protein homolog OS |  |  |  | *Trichoderma harzianum* PE | 25.95 | 3 |
| P33297 | 26S protease regulatory subunit 6A OS | protein catabolic process | Nucleotide binding | Cytoplasm, Nucleus, Proteasome | *Saccharomyces cerevisiae* | 5.07 | 2 |
| P78578 | 26S protease regulatory subunit 6B homolog OS | protein catabolic process | Nucleotide-binding | Cytoplasm, Nucleus, Proteasome | *Aspergillus niger* | 21.28 | 5 |
| Q8SQK0 | 26S protease regulatory subunit 8 homolog OS | protein catabolic process | Nucleotide-binding | Cytoplasm, Nucleus, Proteasome | *Encephalitozoon cuniculi* | 7.91 | 2 |
| Q8WZY4 | 26S proteasome regulatory subunit rpn-8 OS |  |  | Proteasome | *Neurospora crassa* | 7.10 | 2 |
| Q9TEM3 | 2-methylcitrate synthase, mitochondrial OS | carbohydrate metabolic process | Transferase | Mitochondrion | *Aspergillus nidulans* | 9.13 | 4 |
| Q5BCG1 | 3'(2'),5'-bisphosphate nucleotidase OS | Stress response | metal ion binding |  | *Aspergillus nidulans* | 9.09 | 3 |
| P55251 | 3-isopropylmalate dehydratase OS | Amino-acid biosynthesis | Lyase | 3-isopropylmalate dehydratase complex | *Rhizomucor pusillus* | 9.40 | 6 |
| P87256 | 3-isopropylmalate dehydrogenase A OS | Amino-acid biosynthesis | Oxidoreductase | Cytoplasm | *Aspergillus niger* | 16.25 | 4 |
| A2QE32 | 40S ribosomal protein S0 OS | [translation](http://www.ebi.ac.uk/QuickGO/GTerm?id=GO:0006412) | Ribonucleoprotein | Cytoplasm | *Aspergillus niger* | 21.48 | 5 |
| A2R9S1 | 40S ribosomal protein S1 OS | [translation](http://www.ebi.ac.uk/QuickGO/GTerm?id=GO:0006412) | Ribonucleoprotein | Cytoplasm | *Aspergillus niger* | 23.83 | 5 |
| O59936 | 40S ribosomal protein S12 OS | [translation](http://www.ebi.ac.uk/QuickGO/GTerm?id=GO:0006412) | Ribonucleoprotein | ribosome | *Erysiphe graminis* subsp. *hordei* | 8.33 | 2 |
| P19115 | 40S ribosomal protein S14 OS | [translation](http://www.ebi.ac.uk/QuickGO/GTerm?id=GO:0006412) | Ribonucleoprotein | ribosome | *Neurospora crassa* | 20.67 | 2 |
| P27770 | 40S ribosomal protein S17 OS | [translation](http://www.ebi.ac.uk/QuickGO/GTerm?id=GO:0006412) | Ribonucleoprotein | ribosome | *Neurospora crassa* | 21.23 | 3 |
| Q4WI01 | 40S ribosomal protein S21 OS | [translation](http://www.ebi.ac.uk/QuickGO/GTerm?id=GO:0006412) | Ribonucleoprotein | Cytoplasm | *Aspergillus fumigata* | 17.05 | 2 |
| Q7RV75 | 40S ribosomal protein S22 OS | [translation](http://www.ebi.ac.uk/QuickGO/GTerm?id=GO:0006412) | Ribonucleoprotein | ribosome | *Neurospora crassa* | 16.92 | 2 |
| Q873W8 | 40S ribosomal protein S23 OS | [translation](http://www.ebi.ac.uk/QuickGO/GTerm?id=GO:0006412) | Ribonucleoprotein | ribosome | *Aspergillus fumigata* | 12.41 | 2 |
| Q7RVI1 | 40S ribosomal protein S5 OS | [translation](http://www.ebi.ac.uk/QuickGO/GTerm?id=GO:0006412) | Ribonucleoprotein | ribosome | *Neurospora crassa* | 24.88 | 4 |
| O14277 | 40S ribosomal protein S5-A OS | [translation](http://www.ebi.ac.uk/QuickGO/GTerm?id=GO:0006412) | Ribonucleoprotein | [Cytoplasm](http://www.uniprot.org/keywords/KW-0963) | *Schizosaccharomyces pombe* | 15.76 | 2 |
| Q09757 | 40S ribosomal protein S9-A OS | [translation](http://www.ebi.ac.uk/QuickGO/GTerm?id=GO:0006412) | Ribonucleoprotein | [Cytoplasm](http://www.uniprot.org/keywords/KW-0963) | *Schizosaccharomyces pombe* | 8.90 | 2 |
| P14010 | 4-aminobutyrate aminotransferase OS | amide catabolic process | Transferase | Cytoplasm | *Aspergillus nidulans* | 14.66 | 5 |
| P82610 | 5-methyltetrahydropteroyltriglutamate--homocysteine methyltransferase OS | [Amino-acid biosynthesis](http://www.uniprot.org/keywords/KW-0028) | Transferase | [cell surface](http://www.ebi.ac.uk/QuickGO/GTerm?id=GO:0009986) | *Candida albicans* | 2.48 | 2 |
| O66194 | 60 kDa chaperonin (Fragment) OS | [protein refolding](http://www.ebi.ac.uk/QuickGO/GTerm?id=GO:0042026) | Chaperone | [Cytoplasm](http://www.uniprot.org/keywords/KW-0963) | *Enterobacter gergoviae* | 11.11 | 2 |
| A5W8M6 | 60 kDa chaperonin OS | protein refolding | Chaperone | [Cytoplasm](http://www.uniprot.org/keywords/KW-0963) | *Pseudomonas putida* | 20.33 | 6 |
| A6TH53 | 60 kDa chaperonin OS | protein refolding | Chaperone | [Cytoplasm](http://www.uniprot.org/keywords/KW-0963) | *Klebsiella pneumoniae* subsp. *pneumoniae* | 14.96 | 2 |
| O74864 | 60S acidic ribosomal protein P0 OS | cytoplasmic translational elongation | Ribonucleoprotein | cytosol | *Schizosaccharomyces pombe* | 8.01 | 2 |
| Q96TJ5 | 60S acidic ribosomal protein P0 OS | cytoplasmic translational elongation | Ribonucleoprotein | cytosol | *Neurospora crassa* | 7.03 | 2 |
| Q7RZS0 | 60S ribosomal protein L10a OS | translation | Ribonucleoprotein | large ribosomal subunit | *Neurospora crassa* | 9.68 | 2 |
| O13418 | 60S ribosomal protein L15 OS | translation | Ribonucleoprotein | ribosome | *Aspergillus niger* | 12.81 | 2 |
| O74895 | 60S ribosomal protein L15-A OS | translation | Ribonucleoprotein | cytosolic large ribosomal subunit | *Schizosaccharomyces pombe* | 11.94 | 2 |
| Q6MY48 | 60S ribosomal protein L17 OS | translation | Ribonucleoprotein | large ribosomal subunit | *Aspergillus fumigata* | 15.46 | 3 |
| Q8TFH1 | 60S ribosomal protein L18-B OS | translation | Ribonucleoprotein | cytosolic large ribosomal subunit | *Schizosaccharomyces pombe* | 13.37 | 2 |
| Q02753 | 60S ribosomal protein L21-A OS | translation | Ribonucleoprotein | Cytoplasm | *Saccharomyces cerevisiae* | 10.63 | 2 |
| Q8NKF4 | 60S ribosomal protein L3 OS | translation | Ribonucleoprotein | Cytoplasm | *Aspergillus fumigata* | 15.56 | 5 |
| P35679 | 60S ribosomal protein L4-A OS | translation | Ribonucleoprotein | cytosolic large ribosomal subunit | *Schizosaccharomyces pombe* | 7.71 | 2 |
| O59953 | 60S ribosomal protein L5 OS | translation | Ribonucleoprotein | ribosome | *Neurospora crassa* | 6.64 | 2 |
| P38720 | 6-phosphogluconate dehydrogenase, decarboxylating 1 OS | Carbohydrate degradationzation | Oxidoreductase | cytoplasm | *Saccharomyces cerevisiae* | 12.68 | 3 |
| P43774 | 6-phosphogluconate dehydrogenase, decarboxylating OS | Carbohydrate degradation | Oxidoreductase | cytoplasm | *Haemophilus influenzae* | 4.13 | 2 |
| P59769 | 78 kDa glucose-regulated protein homolog OS | response to stress | Chaperone | Endoplasmic reticulum | *Aspergillus awamori* | 24.55 | 11 |
| Q6FW50 | 78 kDa glucose-regulated protein homolog OS | response to stress | Chaperone | Endoplasmic reticulum | *Candida glabrata* | 9.30 | 2 |
| Q9HG01 | 78 kDa glucose-regulated protein homolog OS | response to stress | Chaperone | Endoplasmic reticulum | *Pichia angusta* | 10.83 | 2 |
| P15937 | Acetyl-CoA hydrolase OS | acetyl-CoA metabolic process | Hydrolase | [Cytoplasm](http://www.uniprot.org/keywords/KW-0963) | *Neurospora crassa* | 4.95 | 2 |
| P52910 | Acetyl-coenzyme A synthetase 2 OS | **carbon utilization** | Ligase | Cytoplasm, Nucleus | *Saccharomyces cerevisiae* | 4.39 | 2 |
| P16928 | Acetyl-coenzyme A synthetase OS | **carbon utilization** | Ligase |  | *Aspergillus nidulans* | 7.46 | 5 |
| Q01576 | Acetyl-coenzyme A synthetase OS | **carbon utilization** | Ligase |  | *Phycomyces blakesleeanus* | 4.32 | 3 |
| Q15YI8 | Acetyl-coenzyme A synthetase OS | **carbon utilization** | Ligase |  | *Pseudoalteromonas atlantica* | 3.56 | 2 |
| P19414 | Aconitate hydratase, mitochondrial OS | Carbohydrate metabolism | Lyase | Mitochondrion. Cytoplasm | *Saccharomyces cerevisiae* | 5.53 | 2 |
| O13419 | Actin OS |  | Nucleotide-binding | Cytoplasm, Cytoskeleton | *Botryotinia fuckeliana* | 37.87 | 5 |
| Q99023 | Actin OS |  | Nucleotide-binding | Cytoplasm, Cytoskeleton | *Hypocrea jecorina* | 32.71 | 3 |
| P26197 | Actin-2 OS |  | Nucleotide-binding | Cytoplasm, Cytoskeleton | *Absidia glauca* | 28.12 | 2 |
| O13639 | Adenosylhomocysteinase OS | One-carbon metabolism | Hydrolase | Cytoplasm | *Schizosaccharomyces pombe* | 12.70 | 3 |
| P39954 | Adenosylhomocysteinase OS | One-carbon metabolism | Hydrolase | Cytoplasm | *Saccharomyces cerevisiae* | 8.24 | 2 |
| Q01VU1 | Adenosylhomocysteinase OS | One-carbon metabolism | Hydrolase | Cytoplasm | *Solibacter usitatus* | 4.63 | 2 |
| Q8Y387 | Adenosylhomocysteinase OS | One-carbon metabolism | Hydrolase | Cytoplasm | *Ralstonia solanacearum* | 4.43 | 2 |
| A2QPN9 | Adenylate kinase OS | ADP biosynthetic process | Kinase, Transferase | Cytoplasm, Mitochondrion | *Aspergillus niger* | 15.12 | 3 |
| A2QB52 | Adenylosuccinate synthetase OS | Purine biosynthesis | Ligase | Cytoplasm | *Aspergillus niger* | 14.86 | 5 |
| Q12657 | Adenylyl-sulfate kinase OS | Amino-acid biosynthesis | Kinase, Transferase |  | *Penicillium chrysogenum* PE | 12.80 | 2 |
| P02723 | ADP,ATP carrier protein OS | transport | transporter activity | Mitochondrion inner membrane | *Neurospora crassa* | 17.57 | 3 |
| P49382 | ADP,ATP carrier protein OS | transport | transporter activity | Mitochondrion inner membrane | *Kluyveromyces lactis* | 16.72 | 2 |
| P36579 | ADP-ribosylation factor 1 OS | Transport | Nucleotide-binding | Golgi apparatus | *Schizosaccharomyces pombe* | 23.89 | 2 |
| P34727 | ADP-ribosylation factor OS | Transport | Nucleotide-binding | Golgi apparatus | *Ajellomyces capsulatus* | 23.50 | 4 |
| Q5AQL1 | Alanine--tRNA ligase OS | Protein biosynthesis | Ligase | Mitochondrion | *Aspergillus nidulans* | 2.50 | 2 |
| P41747 | Alcohol dehydrogenase 1 OS | single-organism metabolic process | Oxidoreductase | Cytoplasm | *Aspergillus flavus* | 6.00 | 2 |
| P41751 | Aldehyde dehydrogenase OS | single-organism metabolic process | Oxidoreductase | Cytoplasm | *Aspergillus niger* | 48.89 | 19 |
| B8N106 | Alkaline protease 1 OS | proteolysis | Hydrolase, Protease | extracellular region，Secreted | *Aspergillus flavus* | 11.66 | 3 |
| O74932 | Alpha,alpha-trehalose-phosphate synthase [UDP-forming] OS | trehalose biosynthetic process | Transferase |  | *Yarrowia lipolytica* | 4.26 | 2 |
| O42814 | Alpha-glucuronidase A OS | Carbohydrate metabolism, Polysaccharide degradation, Xylan degradation | Glycosidase, Hydrolase | [Secreted](http://www.uniprot.org/keywords/KW-0964) | *Aspergillus tubingensis* | 2.73 | 2 |
| Q8NK90 | Alpha-N-arabinofuranosidase A OS | Carbohydrate metabolism Polysaccharide degradation | Glycosidase, Hydrolase | [Secreted](http://www.uniprot.org/keywords/KW-0964) | *Aspergillus kawachii* | 9.71 | 5 |
| P42255 | Alpha-N-arabinofuranosidase B OS | Carbohydrate metabolism Polysaccharide degradation | Glycosidase, Hydrolase | Secreted | *Aspergillus niger* | 14.43 | 5 |
| Q8NK89 | Alpha-N-arabinofuranosidase B OS | Carbohydrate metabolism Polysaccharide degradation | Glycosidase, Hydrolase | Secreted | *Aspergillus kawachii* | 27.66 | 8 |
| O42652 | Aspartate aminotransferase, cytoplasmic OS | aspartate metabolic process | Transferase | [cytoplasm](http://www.ebi.ac.uk/QuickGO/GTerm?id=GO:0005737) | *Schizosaccharomyces pombe* | 4.89 | 2 |
| A2R3L3 | Aspartic protease pep1 OS | proteolysis | Aspartyl protease, Hydrolase, Protease | [Secreted](http://www.uniprot.org/keywords/KW-0964) | *Aspergillus niger* | 15.99 | 5 |
| Q2UPZ7 | Aspartyl aminopeptidase OS | proteolysis | Hydrolase, Metalloprotease, Protease |  | *Aspergillus oryzae* | 7.83 | 3 |
| Q870C4 | ATP synthase subunit 4, mitochondrial OS | Transport | Transport | Mitochondrion, Mitochondrion inner membrane | *Paracoccidioides brasiliensis* | 9.84 | 2 |
| Q9P602 | ATP synthase subunit 5, mitochondrial OS | Transport | Hydrolase | [mitochondrial proton-transporting ATP synthase complex](http://www.ebi.ac.uk/QuickGO/GTerm?id=GO:0000276) | *Neurospora crassa* | 9.09 | 2 |
| A6WXW9 | ATP synthase subunit alpha OS | Transport | Hydrolase | [Membrane](http://www.uniprot.org/keywords/KW-0472) | *Ochrobactrum anthropi* | 8.06 | 2 |
| P07251 | ATP synthase subunit alpha, mitochondrial OS | Transport | proton-transporting ATP synthase activity | Mitochondrion inner membrane | *Saccharomyces cerevisiae* | 11.38 | 2 |
| P37211 | ATP synthase subunit alpha, mitochondrial OS | Transport | proton-transporting ATP synthase activity | Mitochondrion inner membrane | *Neurospora crassa* | 9.80 | 3 |
| A5V3X5 | ATP synthase subunit beta OS | Transport | Hydrolase | Membrane | *Sphingomonas wittichii* | 20.87 | 3 |
| P23704 | ATP synthase subunit beta, mitochondrial OS | Transport | Hydrolase | Mitochondrion inner membrane | *Neurospora crassa* | 31.21 | 4 |
| P49376 | ATP synthase subunit beta, mitochondrial OS | Transport | Hydrolase | Mitochondrion inner membrane | *Kluyveromyces lactis* | 35.05 | 5 |
| P0C2C8 | ATP synthase subunit d, mitochondrial OS | Transport | Hydrolase | Mitochondrion inner membrane | *Aspergillus terreus* | 27.17 | 3 |
| O93988 | ATP-citrate synthase subunit 1 OS | [Lipid metabolism](http://www.uniprot.org/keywords/KW-0443) | Transferase | [cytoplasm](http://www.ebi.ac.uk/QuickGO/GTerm?id=GO:0005737) | *Sordaria macrospora* | 4.15 | 2 |
| P15108 | ATP-dependent molecular chaperone HSC82 OS | [Stress response](http://www.uniprot.org/keywords/KW-0346) | Chaperone | Cytoplasm, Mitochondrion | *Saccharomyces cerevisiae* | 9.79 | 2 |
| A1DGZ7 | ATP-dependent RNA helicase dbp2 OS | Ribosome biogenesis, rRNA processing | Helicase, Hydrolase | Cytoplasm | *Aspergillus fischeri* | 5.14 | 3 |
| Q0CLJ6 | ATP-dependent RNA helicase ded1 OS | [Protein biosynthesis](http://www.uniprot.org/keywords/KW-0648) | Helicase, Hydrolase, Initiation factor | Cytoplasm | *Aspergillus terreus* | 9.79 | 5 |
| A2QEN5 | ATP-dependent RNA helicase eIF4A OS | [Protein biosynthesis](http://www.uniprot.org/keywords/KW-0648) | Helicase, Hydrolase, Initiation factor | Cytoplasm | *Aspergillus niger* | 29.40 | 9 |
| Q4P331 | ATP-dependent RNA helicase eIF4A OS | [Protein biosynthesis](http://www.uniprot.org/keywords/KW-0648) | Helicase, Hydrolase, Initiation factor | Cytoplasm | *Ustilago maydis* | 8.27 | 2 |
| A2R0B5 | ATP-dependent RNA helicase sub2 OS | [mRNA processing](http://www.uniprot.org/keywords/KW-0507) | Helicase, Hydrolase | Cytoplasm | *Aspergillus niger* | 12.95 | 4 |
| Q0CGJ9 | ATP-dependent RNA helicase sub2 OS | [mRNA processing](http://www.uniprot.org/keywords/KW-0507) | Helicase, Hydrolase | Cytoplasm | *Aspergillus terreus* | 15.07 | 5 |
| P87076 | Beta-glucosidase A OS | [Carbohydrate metabolism, Cellulose degradation, Polysaccharide degradation](http://www.uniprot.org/keywords/KW-0624) | Glycosidase, Hydrolase | [Secreted](http://www.uniprot.org/keywords/KW-0964) | *Aspergillus kawachii* | 32.09 | 22 |
| P54113 | Bifunctional purine biosynthesis protein ADE16 OS | [Purine biosynthesis](http://www.uniprot.org/keywords/KW-0658) | Hydrolase, Transferase | [cytosol](http://www.ebi.ac.uk/QuickGO/GTerm?id=GO:0005829) | *Saccharomyces cerevisiae* | 3.38 | 2 |
| P41041 | Calmodulin OS |  | calcium ion binding |  | *Pneumocystis carinii* PE | 18.54 | 2 |
| P60206 | Calmodulin OS |  | calcium ion binding |  | *Ajellomyces capsulata* | 61.74 | 9 |
| Q9C196 | cAMP-dependent protein kinase regulatory subunit OS | regulation of protein phosphorylation | cAMP-dependent protein kinase regulator activity | cAMP-dependent protein kinase complex | *Aspergillus niger* | 14.84 | 5 |
| P52719 | Carboxypeptidase cpdS OS | [proteolysis](http://www.ebi.ac.uk/QuickGO/GTerm?id=GO:0006508) | Carboxypeptidase, Hydrolase, Protease |  | *Aspergillus saitoi* | 4.02 | 2 |
| A5AB21 | Carboxypeptidase Y homolog A OS | proteolysis | Carboxypeptidase, Hydrolase, Protease | vacuole | *Aspergillus niger* | 14.36 | 5 |
| C4JNM2 | Carboxypeptidase Y homolog A OS | proteolysis | Carboxypeptidase, Hydrolase, Protease | vacuole | *Uncinocarpus reesii* | 3.33 | 2 |
| P78574 | Catalase A OS | [Hydrogen peroxide](http://www.uniprot.org/keywords/KW-0376) | Peroxidase | [Cytoplasm](http://www.uniprot.org/keywords/KW-0963) | *Aspergillus fumigata* | 3.33 | 2 |
| Q877A8 | Catalase B OS | [Hydrogen peroxide](http://www.uniprot.org/keywords/KW-0376) | Peroxidase | [Secreted](http://www.uniprot.org/keywords/KW-0964) | *Aspergillus oryzae* | 12.83 | 7 |
| P55303 | Catalase R OS | Hydrogen peroxide | Peroxidase |  | *Aspergillus niger* | 4.38 | 2 |
| A2Q7T1 | Catalase-peroxidase OS | Hydrogen peroxide | Peroxidase | Cytoplasm, Secreted | *Aspergillus niger* | 12.34 | 8 |
| P25694 | Cell division control protein 48 OS | Cell cycle, Protein transport, Stress response, Transport | [ATP binding](http://www.ebi.ac.uk/QuickGO/GTerm?id=GO:0005524) | Endoplasmic reticulum, Microsome | *Saccharomyces cerevisiae* | 6.11 | 2 |
| Q5AWS6 | Cell division control protein 48 OS | Cell cycle, Protein transport, Stress response, Transport | ATP binding |  | *Aspergillus nidulans* | 28.43 | 17 |
| A4J7F3 | Chaperone protein DnaK OS | Stress response | Chaperone |  | *Desulfotomaculum reducens* | 5.69 | 2 |
| A6T4F4 | Chaperone protein DnaK OS | Stress response | Chaperone |  | *Klebsiella pneumoniae* subsp. *pneumoniae* | 6.58 | 2 |
| Q0VST6 | Chaperone protein DnaK OS | Stress response | Chaperone |  | *Alcanivorax borkumensis* | 4.06 | 2 |
| Q7NDH1 | Chaperone protein DnaK OS | Stress response | Chaperone |  | *Gloeobacter violaceus* | 4.08 | 2 |
| P51044 | Citrate synthase, mitochondrial OS | Tricarboxylic acid cycle | Transferase | [Mitochondrion](http://www.uniprot.org/keywords/KW-0496) | *Aspergillus niger* | 33.47 | 12 |
| A2QDB9 | Clustered mitochondria protein homolog OS | intracellular distribution of mitochondria |  | [Cytoplasm](http://www.uniprot.org/keywords/KW-0963) | *Aspergillus niger* | 3.52 | 4 |
| B0XV83 | Cyanate hydratase OS | cyanate metabolic process | Lyase |  | *Aspergillus fumigata* | 35.71 | 4 |
| P00046 | Cytochrome c OS | Electron transport, Respiratory chain, Transport | electron transporter, | Mitochondrion | *Schizosaccharomyces pombe* | 16.51 | 2 |
| P19681 | Cytochrome c OS | Electron transport, Respiratory chain, Transport | electron carrier activity | Mitochondrion | *Schwanniomyces occidentalis* | 23.64 | 2 |
| P38091 | Cytochrome c OS | Electron transport, Respiratory chain, Transport | electron carrier activity | Mitochondrion | *Aspergillus nidulans* | 30.09 | 2 |
| P56205 | Cytochrome c OS | Electron transport, Respiratory chain, Transport | electron carrier activity | Mitochondrion | *Aspergillus niger* | 40.54 | 3 |
| O93980 | Cytochrome c oxidase polypeptide 5, mitochondrial OS | oxidation-reduction process | Oxidoreductase |  | *Aspergillus niger* | 12.18 | 2 |
| P0C0V3 | Cytochrome c peroxidase, mitochondrial OS | cytochrome-c peroxidase activity | Oxidoreductase, Peroxidase | Mitochondrion | *Aspergillus nidulans* | 8.86 | 2 |
| Q4WPF8 | Cytochrome c peroxidase, mitochondrial OS | response to oxidative stress | Oxidoreductase, Peroxidase | Mitochondrion | *Aspergillus fumigata* | 15.03 | 3 |
| Q9P8I0 | Delta-1-pyrroline-5-carboxylate dehydrogenase, mitochondrial OS | Proline metabolism | Oxidoreductase | [Mitochondrion](http://www.uniprot.org/keywords/KW-0496) | *Aspergillus nidulans* | 9.44 | 4 |
| P0A1R7 | DNA-binding protein HU-alpha OS | DNA condensation | DNA binding |  | *Salmonella typhi* | 31.11 | 2 |
| P05384 | DNA-binding protein HU-beta OS | DNA condensation | DNA binding |  | *Pseudomonas aeruginosa* | 32.22 | 2 |
| P28295 | Elongation factor 1-alpha OS | Protein biosynthesis | Elongation factor | [Cytoplasm](http://www.uniprot.org/keywords/KW-0963) | *Absidia glauca* | 22.71 | 4 |
| Q9Y713 | Elongation factor 1-alpha OS | Protein biosynthesis | Elongation factor | Cytoplasm | *Aspergillus oryzae* | 13.91 | 2 |
| O14460 | Elongation factor 2 OS | Protein biosynthesis | Elongation factor | Cytoplasm | *Schizosaccharomyces pombe* | 3.80 | 2 |
| Q96X45 | Elongation factor 2 OS | Protein biosynthesis | Elongation factor | Cytoplasm | *Neurospora crassa* | 5.69 | 4 |
| A5U9R0 | Elongation factor G OS | Protein biosynthesis | Elongation factor | Cytoplasm | *Haemophilus influenzae* | 3.71 | 2 |
| A6TEX7 | Elongation factor Tu OS | Protein biosynthesis | Elongation factor | Cytoplasm | *Klebsiella pneumoniae* subsp. *pneumoniae* | 21.83 | 3 |
| B0TC54 | Elongation factor Tu OS | Protein biosynthesis | Elongation factor | Cytoplasm | *Heliobacterium modesticaldum* | 7.25 | 2 |
| P02992 | Elongation factor Tu, mitochondrial OS | Protein biosynthesis | [Oxidoreductase](http://www.uniprot.org/keywords/KW-0560) | Cytoplasm | *Saccharomyces cerevisiae* | 9.38 | 3 |
| P40952 | Enoate reductase 1 OS | Protein biosynthesis | Oxidoreductase |  | *Kluyveromyces lactis* | 5.53 | 2 |
| P42894 | Enolase OS | Glycolysis | Lyase | Cytoplasm | *Neocallimastix frontalis PE* | 7.34 | 2 |
| Q12560 | Enolase OS | Glycolysis | Lyase | Cytoplasm | *Aspergillus oryzae* | 38.81 | 2 |
| Q96X46 | Enolase OS | Glycolysis | Lyase | Cytoplasm | *Penicillium citrinum* | 39.27 | 2 |
| A1JJR4 | Enolase OS | Glycolysis | Lyase | Cytoplasm | *Yersinia enterocolitica* serotype O:8 / biotype 1B | 9.28 | 3 |
| A2Q908 | Eukaryotic translation initiation factor 3 subunit B OS | Protein biosynthesis | Initiation factor | Cytoplasm | *Aspergillus niger* | 2.97 | 2 |
| Q5BGH1 | Eukaryotic translation initiation factor 3 subunit B OS | Protein biosynthesis | Initiation factor | Cytoplasm | *Aspergillus nidulans* | 2.71 | 2 |
| Q2UKS9 | Eukaryotic translation initiation factor 3 subunit E OS | Protein biosynthesis | Initiation factor | Cytoplasm | *Aspergillus oryzae* | 12.42 | 5 |
| A2QQ10 | Eukaryotic translation initiation factor 3 subunit F OS | Protein biosynthesis | Initiation factor | Cytoplasm | *Aspergillus niger* | 12.27 | 4 |
| A2QQA2 | Eukaryotic translation initiation factor 3 subunit H OS | Protein biosynthesis | Initiation factor | Cytoplasm | *Aspergillus niger* | 11.51 | 4 |
| A2QEV8 | Eukaryotic translation initiation factor 3 subunit I OS | Protein biosynthesis | Initiation factor | Cytoplasm | *Aspergillus niger* | 10.98 | 3 |
| Q2UQ34 | Eukaryotic translation initiation factor 3 subunit I OS | Protein biosynthesis | Initiation factor | Cytoplasm | *Aspergillus oryzae* | 5.97 | 2 |
| A2R7B4 | Eukaryotic translation initiation factor 3 subunit K OS | Protein biosynthesis | Initiation factor | Cytoplasm | *Aspergillus niger* | 8.24 | 2 |
| A1C690 | Eukaryotic translation initiation factor 3 subunit L OS | Protein biosynthesis | Initiation factor | Cytoplasm | *Aspergillus clavatus* | 6.09 | 2 |
| Q0CPV5 | Eukaryotic translation initiation factor 3 subunit M OS | Protein biosynthesis | Initiation factor | Cytoplasm | *Aspergillus terreus* | 5.21 | 2 |
| Q2UHU9 | F-actin-capping protein subunit alpha OS | actin cytoskeleton organization | Actin capping | Cytoplasm, Cytoskeleton | *Aspergillus oryzae* | 12.82 | 3 |
| Q2URJ3 | F-actin-capping protein subunit beta OS | actin cytoskeleton organization | Actin capping | Cytoplasm, Cytoskeleton | *Aspergillus oryzae* | 8.65 | 2 |
| Q4WLV6 | FK506-binding protein 1A OS | protein folding | Isomerase, Rotamase | cytoplasm | *Aspergillus fumigata* | 23.21 | 2 |
| Q07103 | Formate dehydrogenase OS | oxidation-reduction process | Oxidoreductase | cytosol | *Neurospora crassa* | 4.53 | 2 |
| A2QI68 | Formation of crista junctions protein 1 OS |  |  | Mitochondrion inner membrane; Single-pass membrane protein | *Aspergillus niger* | 9.51 | 4 |
| Q9HGY9 | Fructose-bisphosphate aldolase OS | [Glycolysis](http://www.uniprot.org/keywords/KW-0324) | Lyase |  | *Aspergillus oryzae* | 12.15 | 2 |
| Q00770 | Fumarylacetoacetase OS | Phenylalanine catabolism, Tyrosine catabolism | Hydrolase | intracellular | *Aspergillus nidulans* | 3.94 | 2 |
| P22832 | Glucoamylase OS | Carbohydrate metabolism,Polysaccharide degradation |  |  | *Aspergillus shirousami* | 8.76 | 4 |
| Q92407 | Glucokinase OS | glycolysis | Kinase, Transferase |  | *Aspergillus niger* | 12.32 | 5 |
| P48826 | Glucose-6-phosphate 1-dehydrogenase OS | Carbohydrate metabolism, Glucose metabolism | Oxidoreductase |  | *Aspergillus niger* | 29.61 | 13 |
| Q9HGZ2 | Glucose-6-phosphate isomerase OS | Gluconeogenesis, Glycolysis | Isomerase | [cytoplasm](http://www.ebi.ac.uk/QuickGO/GTerm?id=GO:0005737) | *Aspergillus oryzae* | 25.86 | 8 |
| P37902 | Glutamate/aspartate periplasmic-binding protein OS | Transport | transporter activity | [Periplasm](http://www.uniprot.org/keywords/KW-0574) | *Escherichia coli* | 13.25 | 3 |
| Q9C136 | Glyceraldehyde-3-phosphate dehydrogenase 1 OS | Glycolysis | Oxidoreductase | [cytoplasm](http://www.ebi.ac.uk/QuickGO/GTerm?id=GO:0005737) | *Mucor circinelloides* f. *lusitanicus* | 8.61 | 2 |
| B7LQ20 | Glyceraldehyde-3-phosphate dehydrogenase A OS | Glycolysis | Oxidoreductase | [cytoplasm](http://www.ebi.ac.uk/QuickGO/GTerm?id=GO:0005737) | *Escherichia fergusonii* | 18.73 | 5 |
| P35143 | Glyceraldehyde-3-phosphate dehydrogenase OS | Glycolysis | Oxidoreductase | [cytoplasm](http://www.ebi.ac.uk/QuickGO/GTerm?id=GO:0005737) | *Colletotrichum gloeosporioides* | 21.89 | 2 |
| Q12552 | Glyceraldehyde-3-phosphate dehydrogenase OS | Glycolysis | Oxidoreductase | [cytoplasm](http://www.ebi.ac.uk/QuickGO/GTerm?id=GO:0005737) | *Aspergillus niger* | 63.10 | 13 |
| Q8NK47 | Glyceraldehyde-3-phosphate dehydrogenase OS | Glycolysis | Oxidoreductase | [cytoplasm](http://www.ebi.ac.uk/QuickGO/GTerm?id=GO:0005737) | *Rhizomucor miehei* | 28.57 | 6 |
| Q74ZA9 | GTP-binding nuclear protein GSP1/Ran OS | Protein transport, Transport | intracellular protein transport | [nucleus](http://www.ebi.ac.uk/QuickGO/GTerm?id=GO:0005634) | *Ashbya gossypii* | 23.36 | 5 |
| Q9C3Y4 | GTP-binding protein rhoA OS | hyphal growth | GTP binding | Cell membrane, Membrane | *Aspergillus nidulans* | 11.92 | 2 |
| P33723 | GTP-binding protein ypt1 OS | Protein transport, Transport | protein transport | Cell membrane, Membrane | *Neurospora crassa* | 34.48 | 6 |
| P51996 | GTP-binding protein YPT32/YPT11 OS | Autophagy, Exocytosis, Protein transport, Transport | GTP binding | Golgi membrane | *Saccharomyces cerevisiae* | 15.32 | 2 |
| P32939 | GTP-binding protein YPT7 OS | Protein transport, Transport | GTP binding | [Vacuole](http://www.uniprot.org/keywords/KW-0926) | *Saccharomyces cerevisiae* | 14.90 | 2 |
| Q9HDY0 | GTP-binding protein ypt71 OS | Protein transport, Transport | GTP binding | [Vacuole](http://www.uniprot.org/keywords/KW-0926) | *Schizosaccharomyces pombe* | 10.10 | 2 |
| Q01369 | Guanine nucleotide-binding protein subunit beta-like protein OS |  |  |  | *Neurospora crassa* | 6.96 | 2 |
| P18694 | Heat shock 70 kDa protein 2 OS | [Stress response](http://www.uniprot.org/keywords/KW-0346) | ATP binding | Mitochondrion | *Ustilago maydis* | 13.33 | 2 |
| Q5B0C0 | Heat shock 70 kDa protein OS | [Stress response](http://www.uniprot.org/keywords/KW-0346) | ATP binding | Mitochondrion | *Aspergillus nidulans* | 18.17 | 10 |
| Q5B2V1 | Heat shock 70 kDa protein OS | [Stress response](http://www.uniprot.org/keywords/KW-0346) | ATP binding | Mitochondrion | *Aspergillus nidulans* | 28.11 | 3 |
| Q5B041 | Heat shock protein 60 OS | [Stress response](http://www.uniprot.org/keywords/KW-0346) | ATP binding | Mitochondrion | *Aspergillus nidulans* | 38.78 | 8 |
| O74261 | Heat shock protein 60, mitochondrial OS | [Stress response](http://www.uniprot.org/keywords/KW-0346) | Chaperone | Mitochondrion | *Candida albicans* | 12.90 | 2 |
| O43109 | Heat shock protein 90 homolog OS | [Stress response](http://www.uniprot.org/keywords/KW-0346) | Chaperone | [Cytoplasm](http://www.uniprot.org/keywords/KW-0963) | *Podospora anserina* | 14.12 | 2 |
| P40292 | Heat shock protein 90 OS | [Stress response](http://www.uniprot.org/keywords/KW-0346) | Chaperone | [Cytoplasm](http://www.uniprot.org/keywords/KW-0963) | *Aspergillus fumigata* | 27.20 | 8 |
| Q8J2M3 | Heat shock protein HSP82 OS | [Stress response](http://www.uniprot.org/keywords/KW-0346) | Chaperone | [Cytoplasm](http://www.uniprot.org/keywords/KW-0963) | *Ashbya gossypii* | 8.38 | 2 |
| O74225 | Heat shock protein Hsp88 OS | [Stress response](http://www.uniprot.org/keywords/KW-0346) | ATP binding | [Cytoplasm](http://www.uniprot.org/keywords/KW-0963) | *Neurospora crassa* | 2.83 | 2 |
| P31540 | Heat shock protein hsp98 OS | [Stress response](http://www.uniprot.org/keywords/KW-0346) | ATP binding | Cytoplasm, Nucleus | *Neurospora crassa* | 1.94 | 2 |
| P46587 | Heat shock protein SSA2 OS | [Stress response](http://www.uniprot.org/keywords/KW-0346) | ATP binding | Cell wall, Cytoplasm, Secreted | *Candida albicans* | 12.71 | 2 |
| P41770 | Heat shock protein SSB OS | [Stress response](http://www.uniprot.org/keywords/KW-0346) | ATP binding | Chromosome, Nucleosome core, Nucleus | *Kluyveromyces marxianus* | 6.85 | 2 |
| A1CJ09 | Histone H2B OS | [nucleosome assembly](http://www.ebi.ac.uk/QuickGO/GTerm?id=GO:0006334) | DNA binding | Chromosome, Nucleosome core, Nucleus | *Aspergillus clavatus* | 31.43 | 4 |
| Q8J1K2 | Histone H2B OS | nucleosome assembly | DNA binding | Chromosome, Nucleosome core, Nucleus | *Rosellinia necatrix* | 29.41 | 4 |
| P23750 | Histone H4.1 OS | nucleosome assembly | DNA binding | Chromosome, Nucleosome core, Nucleus | *Aspergillus nidulans* | 40.78 | 2 |
| Q58FL6 | Homoaconitase, mitochondrial OS | [Amino-acid biosynthesis](http://www.uniprot.org/keywords/KW-0028) | [Lyase](http://www.uniprot.org/keywords/KW-0456) | [Mitochondrion](http://www.uniprot.org/keywords/KW-0496) | *Aspergillus niger* | 3.25 | 2 |
| Q5B998 | Homoserine dehydrogenase OS | Amino-acid biosynthesis | Oxidoreductase |  | *Aspergillus nidulans* | 5.43 | 2 |
| Q5B912 | Inorganic pyrophosphatase OS | Stress response | Hydrolase | [Cytoplasm](http://www.uniprot.org/keywords/KW-0963) | *Aspergillus nidulans* | 17.07 | 2 |
| Q6MVH7 | Inorganic pyrophosphatase OS | Stress response | Hydrolase | [Cytoplasm](http://www.uniprot.org/keywords/KW-0963) | *Neurospora crassa* | 21.38 | 2 |
| Q757J8 | Inorganic pyrophosphatase OS | Stress response | Hydrolase | [Cytoplasm](http://www.uniprot.org/keywords/KW-0963) | *Ashbya gossypii* | 8.01 | 2 |
| P08200 | Isocitrate dehydrogenase [NADP] OS | Glyoxylate bypass,Tricarboxylic acid cycle | Oxidoreductase | [cytoplasm](http://www.ebi.ac.uk/QuickGO/GTerm?id=GO:0005737) | *Escherichia coli* | 5.53 | 2 |
| P50215 | Isocitrate dehydrogenase [NADP] OS | Glyoxylate bypass,Tricarboxylic acid cycle | Oxidoreductase | [cytoplasm](http://www.ebi.ac.uk/QuickGO/GTerm?id=GO:0005737) | *Sphingomonas yanoikuyae* | 6.16 | 2 |
| P65098 | Isocitrate dehydrogenase [NADP] OS | Glyoxylate bypass,Tricarboxylic acid cycle | Oxidoreductase | [cytoplasm](http://www.ebi.ac.uk/QuickGO/GTerm?id=GO:0005737) | *Mycobacterium bovis* | 10.02 | 2 |
| P79089 | Isocitrate dehydrogenase [NADP], mitochondrial OS | Glyoxylate bypass,Tricarboxylic acid cycle | Oxidoreductase | [Mitochondrion](http://www.uniprot.org/keywords/KW-0496) | *Aspergillus niger* | 46.18 | 18 |
| Q96WZ5 | Isocitrate lyase OS | Glyoxylate bypass,Tricarboxylic acid cycle | [Lyase](http://www.uniprot.org/keywords/KW-0456) | [Peroxisome](http://www.uniprot.org/keywords/KW-0576) | *Penicillium marneffei* | 7.78 | 3 |
| A8ACS4 | Ketol-acid reductoisomerase OS | [Amino-acid biosynthesis](http://www.uniprot.org/keywords/KW-0028) | [Oxidoreductase](http://www.uniprot.org/keywords/KW-0560) |  | *Citrobacter koseri* | 7.74 | 3 |
| P38674 | Ketol-acid reductoisomerase, mitochondrial OS | [Amino-acid biosynthesis](http://www.uniprot.org/keywords/KW-0028) | [Oxidoreductase](http://www.uniprot.org/keywords/KW-0560) | [Mitochondrion](http://www.uniprot.org/keywords/KW-0496) | *Neurospora crassa* | 10.95 | 2 |
| A2QKF8 | Leukotriene A-4 hydrolase homolog OS | [Leukotriene biosynthesis](http://www.uniprot.org/keywords/KW-0434) | Hydrolase, Metalloprotease, Protease | [cytoplasm](http://www.ebi.ac.uk/QuickGO/GTerm?id=GO:0005737) | *Aspergillus niger* | 3.88 | 2 |
| Q4X0Z7 | Lon protease homolog, mitochondrial OS | [response to oxidative stress](http://www.ebi.ac.uk/QuickGO/GTerm?id=GO:0034599) | Hydrolase, Protease, Serine protease | [mitochondrial matrix](http://www.ebi.ac.uk/QuickGO/GTerm?id=GO:0005759) | *Aspergillus fumigata* | 2.08 | 2 |
| P61894 | Malate dehydrogenase (Fragment) OS | [Tricarboxylic acid cycle](http://www.uniprot.org/keywords/KW-0816) | Oxidoreductase |  | *Klebsiella oxytoca* | 17.49 | 2 |
| A4WF48 | Malate dehydrogenase OS | [Tricarboxylic acid cycle](http://www.uniprot.org/keywords/KW-0816) | Oxidoreductase |  | *Enterobacter* sp. | 16.35 | 2 |
| P83778 | Malate dehydrogenase, cytoplasmic OS | [Tricarboxylic acid cycle](http://www.uniprot.org/keywords/KW-0816) | Oxidoreductase | [Cytoplasm](http://www.uniprot.org/keywords/KW-0963) | *Candida albicans* | 7.12 | 2 |
| P28344 | Malate synthase, glyoxysomal OS | [Glyoxylate bypass,Tricarboxylic acid cycle](http://www.uniprot.org/keywords/KW-0329) | Transferase | Glyoxysome, Peroxisome | *Aspergillus nidulans* | 7.79 | 4 |
| A2QGA1 | Mannitol 2-dehydrogenase OS | [coenzyme binding](http://www.ebi.ac.uk/QuickGO/GTerm?id=GO:0050662) | Oxidoreductase |  | *Aspergillus niger* | 19.26 | 7 |
| A2QD49 | Mannitol-1-phosphate 5-dehydrogenase OS | [cellular response to heat](http://www.ebi.ac.uk/QuickGO/GTerm?id=GO:0034605) | Oxidoreductase |  | *Aspergillus niger* | 12.37 | 2 |
| Q12563 | Mannosyl-oligosaccharide alpha-1,2-mannosidase 1B OS | Carbohydrate metabolism | Glycosidase, Hydrolase | Cytoplasmic vesicle | *Aspergillus saitoi* | 22.61 | 9 |
| P09177 | Mucorpepsin OS |  | Hydrolase, Protease |  | *Rhizomucor pusillus* PE | 21.08 | 7 |
| P31867 | NAD(P)H-dependent D-xylose reductase OS | Carbohydrate metabolism; D-xylose degradation. | [Oxidoreductase](http://www.uniprot.org/keywords/KW-0560) |  | *Scheffersomyces stipitis* | 2.83 | 2 |
| A2Q898 | NADH-cytochrome b5 reductase 2 OS |  | Oxidoreductase | Mitochondrion outer membrane; | *Aspergillus niger* | 28.26 | 6 |
| P22142 | NADH-ubiquinone oxidoreductase 49 kDa subunit, mitochondrial OS | [Transport](http://www.uniprot.org/keywords/KW-0813) | Oxidoreductase | Mitochondrion inner membrane | *Neurospora crassa* | 3.77 | 2 |
| Q92406 | NADH-ubiquinone oxidoreductase 51 kDa subunit, mitochondrial OS | [Transport](http://www.uniprot.org/keywords/KW-0813) | Oxidoreductase | Mitochondrion inner membrane | *Aspergillus niger* | 6.85 | 3 |
| P55804 | NADP(+)-dependent glycerol dehydrogenase (Fragments) OS |  | [Oxidoreductase](http://www.uniprot.org/keywords/KW-0560) |  | *Aspergillus niger* PE | 31.31 | 2 |
| P18819 | NADP-specific glutamate dehydrogenase OS | cellular amino acid metabolic process | [Oxidoreductase](http://www.uniprot.org/keywords/KW-0560) | mitochondrion | *Aspergillus nidulans* | 4.58 | 2 |
| P00365 | NAD-specific glutamate dehydrogenase OS | glutamate catabolic process to 2-oxoglutarate | Oxidoreductase | mitochondrion | *Neurospora crassa* | 1.71 | 2 |
| A2R4V1 | Nascent polypeptide-associated complex subunit alpha OS | [Transport](http://www.uniprot.org/keywords/KW-0813) | Repressor | Cytoplasm, Nucleus | *Aspergillus niger* | 28.22 | 3 |
| A2R091 | Nascent polypeptide-associated complex subunit beta OS | [Transport](http://www.uniprot.org/keywords/KW-0813) | Repressor | Cytoplasm, Nucleus | *Aspergillus niger* | 50.97 | 5 |
| Q8TFN0 | Nucleoside diphosphate kinase OS | [Nucleotide metabolism](http://www.uniprot.org/keywords/KW-0546) | Kinase, Transferase | extracellular region | *Aspergillus nidulans* | 16.99 | 2 |
| Q92413 | Ornithine aminotransferase OS | L-proline biosynthetic process | Transferase | Cytoplasm | *Aspergillus nidulans* | 5.07 | 2 |
| P07817 | Orotidine 5'-phosphate decarboxylase OS | Pyrimidine biosynthesis | Decarboxylase, Lyase |  | *Aspergillus niger* | 24.19 | 6 |
| Q9Y720 | Orotidine 5'-phosphate decarboxylase OS | Pyrimidine biosynthesis | Decarboxylase, Lyase |  | *Rhizomucor pusillus* | 8.37 | 2 |
| P24016 | Outer membrane protein A (Fragment) OS | [Transport](http://www.uniprot.org/keywords/KW-0813) | Porin | Cell outer membrane | *Citrobacter freundii* | 15.13 | 2 |
| Q48473 | Outer membrane protein C OS | [Transport](http://www.uniprot.org/keywords/KW-0813) | Porin | Cell outer membrane | *Klebsiella pneumoniae* | 6.06 | 2 |
| P17872 | Pectinesterase OS | [Cell wall biogenesis/degradation](http://www.uniprot.org/keywords/KW-0961) | Aspartyl esterase, Hydrolase | [Secreted](http://www.uniprot.org/keywords/KW-0964) | *Aspergillus tubingensis* | 8.16 | 2 |
| Q8X166 | Peptidyl-prolyl cis-trans isomerase B OS | [protein folding](http://www.ebi.ac.uk/QuickGO/GTerm?id=GO:0006457) | Isomerase, Rotamase | Cyclosporin | *Aspergillus niger* | 24.06 | 4 |
| Q5B4E7 | Peptidyl-prolyl cis-trans isomerase D OS | [protein folding](http://www.ebi.ac.uk/QuickGO/GTerm?id=GO:0006457) | Isomerase, Rotamase | Cytoplasm | *Aspergillus nidulans* | 12.37 | 4 |
| P22011 | Peptidyl-prolyl cis-trans isomerase OS | [protein refolding](http://www.ebi.ac.uk/QuickGO/GTerm?id=GO:0042026) | Isomerase, Rotamase | Cyclosporin | *Candida albicans* | 17.90 | 2 |
| Q96UL8 | Phosphoenolpyruvate carboxykinase [ATP] OS | [Gluconeogenesis](http://www.uniprot.org/keywords/KW-0312) | Decarboxylase, Lyase |  | *Aspergillus nidulans* | 14.00 | 5 |
| Q9P931 | Phosphoglucomutase OS | [Carbohydrate metabolism](http://www.uniprot.org/keywords/KW-0119) | Isomerase | Cytoplasm | *Aspergillus nidulans* | 17.99 | 7 |
| P14828 | Phosphoglycerate kinase OS | [Glycolysis](http://www.uniprot.org/keywords/KW-0324) | Kinase, Transferase | Cytoplasm | *Kluyveromyces lactis* | 2.40 | 2 |
| P41756 | Phosphoglycerate kinase OS | [Glycolysis](http://www.uniprot.org/keywords/KW-0324) | Kinase, Transferase | Cytoplasm | *Aspergillus oryzae* | 34.05 | 5 |
| A5GMA8 | Photosystem II D2 protein OS | Transport | Oxidoreductase | Cellular thylakoid membrane | *Synechococcus* sp. | 6.55 | 2 |
| Q10VK7 | Photosystem Q(B) protein 2 OS | transport | Oxidoreductase | Membrane | *Trichodesmium erythraeum* | 6.23 | 2 |
| A2Q848 | Polyadenylate-binding protein, cytoplasmic and nuclear OS | [mRNA transport, Translation regulation](http://www.uniprot.org/keywords/KW-0507) | nucleotide binding | Cytoplasm, Nucleus | *Aspergillus niger* | 7.80 | 4 |
| Q5B2Q4 | Probable 1,4-beta-D-glucan cellobiohydrolase A OS | Carbohydrate metabolism,Cellulose degradation,Polysaccharide degradation, | Glycosidase, Hydrolase | [Secreted](http://www.uniprot.org/keywords/KW-0964) | *Aspergillus nidulans* | 4.93 | 2 |
| O74445 | Probable 26S protease subunit rpt4 OS | [ATP catabolic process](http://www.ebi.ac.uk/QuickGO/GTerm?id=GO:0006200) | [ATP binding](http://www.ebi.ac.uk/QuickGO/GTerm?id=GO:0005524) | Proteasome | *Schizosaccharomyces pombe* | 8.51 | 2 |
| Q12428 | Probable 2-methylcitrate dehydratase OS | propionate metabolic process | Lyase |  | *Saccharomyces cerevisiae* | 3.49 | 2 |
| Q9UT19 | Probable 5-methyltetrahydropteroyltriglutamate--homocysteine methyltransferase OS | Amino-acid biosynthesis | Methyltransferase, Transferase | Cytoplasm, Nucleus | *Schizosaccharomyces pombe* | 3.40 | 2 |
| A2R2S6 | Probable alpha-galactosidase D OS | Carbohydrate metabolism,Polysaccharide degradation | Glycosidase, Hydrolase | Secreted | *Aspergillus niger* | 5.30 | 3 |
| Q0CVX4 | Probable alpha-galactosidase D OS | Carbohydrate metabolism,Polysaccharide degradation | Glycosidase, Hydrolase | Secreted | *Aspergillus terreus* | 2.90 | 2 |
| P79021 | Probable alpha-L-arabinofuranosidase axhA OS | Carbohydrate metabolism,Polysaccharide degradation | Glycosidase, Hydrolase | Secreted | *Aspergillus tubingensis* | 14.76 | 4 |
| A5AAG2 | Probable arabinan endo-1,5-alpha-L-arabinosidase C OS | Carbohydrate metabolism,Polysaccharide degradation | Glycosidase, Hydrolase | Secreted | *Aspergillus niger* | 10.38 | 3 |
| A2RB93 | Probable arabinogalactan endo-1,4-beta-galactosidase A OS | Carbohydrate metabolism,Polysaccharide degradation | Glycosidase, Hydrolase | Secreted | *Aspergillus niger* | 7.71 | 2 |
| Q9Y7F8 | Probable arabinogalactan endo-1,4-beta-galactosidase A OS | Carbohydrate metabolism,Polysaccharide degradation | Glycosidase, Hydrolase | Secreted | *Aspergillus tubingensis* | 16.57 | 4 |
| Q8X097 | Probable ATP-citrate synthase subunit 1 OS | Lipid biosynthesis, Lipid metabolism | Transferase | Cytoplasm | *Neurospora crassa* | 9.40 | 5 |
| A2QAN3 | Probable beta-galactosidase A OS | Carbohydrate metabolism,Polysaccharide degradation | Glycosidase, Hydrolase | Secreted | *Aspergillus niger* | 8.54 | 8 |
| A2QA64 | Probable beta-galactosidase B OS | Carbohydrate metabolism,Polysaccharide degradation | Glycosidase, Hydrolase | Secreted | *Aspergillus niger* | 4.82 | 4 |
| A2QL84 | Probable beta-galactosidase C OS | Carbohydrate metabolism,Polysaccharide degradation | Glycosidase, Hydrolase | Secreted | *Aspergillus niger* | 3.02 | 2 |
| A2R989 | Probable beta-glucosidase I OS | Carbohydrate metabolism,Polysaccharide degradation | Glycosidase, Hydrolase | Secreted | *Aspergillus niger* | 4.52 | 3 |
| A5ABF5 | Probable beta-glucosidase M OS | Carbohydrate metabolism,Polysaccharide degradation | Glycosidase, Hydrolase | Secreted | *Aspergillus niger* | 5.88 | 4 |
| A2RBC2 | Probable carboxypeptidase An18g06210 OS | [proteolysis](http://www.ebi.ac.uk/QuickGO/GTerm?id=GO:0006508) | Hydrolase, Protease | Secreted | *Aspergillus niger* | 4.81 | 2 |
| Q10306 | Probable citrate synthase, mitochondrial OS | [Tricarboxylic acid cycle](http://www.uniprot.org/keywords/KW-0816) | Transferase | Mitochondrion | *Schizosaccharomyces pombe* | 4.14 | 2 |
| A2QMS4 | Probable D-xylulose kinase A OS | Carbohydrate metabolism Xylose metabolism | Kinase, Transferase |  | *Aspergillus niger* | 11.93 | 5 |
| A2QY54 | Probable D-xylulose reductase A OS | Carbohydrate metabolism, Xylose metabolism | Oxidoreductase |  | *Aspergillus niger* | 8.10 | 3 |
| A2QBQ3 | Probable endo-1,3(4)-beta-glucanase An02g00850 OS | Carbohydrate metabolism,Polysaccharide degradation | Glycosidase, Hydrolase | Membrane | *Aspergillus niger* | 2.98 | 2 |
| A2Q7I0 | Probable endo-1,4-beta-xylanase B OS | Carbohydrate metabolism,Polysaccharide degradation | Glycosidase, Hydrolase | Secreted | *Aspergillus niger* | 14.67 | 2 |
| A2QFV7 | Probable endo-1,4-beta-xylanase C OS | Carbohydrate metabolism,Polysaccharide degradation | Glycosidase, Hydrolase | Secreted | *Aspergillus niger* | 18.96 | 5 |
| Q96WQ8 | Probable endo-beta-1,4-glucanase B OS | Carbohydrate metabolism, Cellulose degradation, Polysaccharide degradation | Glycosidase, Hydrolase | [Secreted](http://www.uniprot.org/keywords/KW-0964) | *Aspergillus kawachii* | 20.18 | 4 |
| A2R8F8 | Probable endopolygalacturonase A OS | Cell wall biogenesis/degradation | Glycosidase, Hydrolase | [Secreted](http://www.uniprot.org/keywords/KW-0964) | *Aspergillus niger* | 6.49 | 2 |
| A2QK83 | Probable endo-xylogalacturonan hydrolase A OS | Carbohydrate metabolism,Polysaccharide degradation | Glycosidase, Hydrolase | Secreted | *Aspergillus niger* | 8.87 | 3 |
| A2QA27 | Probable exo-1,4-beta-xylosidase xlnD OS | Carbohydrate metabolism,Polysaccharide degradation | Glycosidase, Hydrolase | Secreted | *Aspergillus niger* | 11.44 | 7 |
| A2QHG0 | Probable exopolygalacturonase B OS | Cell wall biogenesis/degradation | Glycosidase, Hydrolase | [Secreted](http://www.uniprot.org/keywords/KW-0964) | *Aspergillus niger* | 5.48 | 2 |
| A2R0Z6 | Probable feruloyl esterase B OS | Carbohydrate metabolism,Polysaccharide degradation | Hydrolase, Serine esterase | Secreted | *Aspergillus niger* | 6.33 | 2 |
| Q03134 | Probable formate dehydrogenase OS | acetate metabolic process | Oxidoreductase | extracellular region | *Aspergillus nidulans* | 4.38 | 2 |
| A2RAR6 | Probable glucan 1,3-beta-glucosidase A OS | Carbohydrate metabolism,Polysaccharide degradation | Glycosidase, Hydrolase | Secreted | *Aspergillus niger* | 15.14 | 6 |
| A2QH21 | Probable glucan endo-1,3-beta-glucosidase eglC OS | Carbohydrate metabolism,Polysaccharide degradation | Hydrolase | Secreted | *Aspergillus niger* | 4.78 | 2 |
| O59855 | Probable heat shock protein ssa2 OS | Stress response | ATP binding | Cytoplasm | *Schizosaccharomyces pombe* | 11.44 | 3 |
| O14254 | Probable isocitrate dehydrogenase [NADP], mitochondrial OS | Tricarboxylic acid cycle | Oxidoreductase | Mitochondrion | *Schizosaccharomyces pombe* | 6.83 | 2 |
| P78827 | Probable ketol-acid reductoisomerase, mitochondrial OS | Amino-acid biosynthesis | Oxidoreductase | Mitochondrion | *Schizosaccharomyces pombe* | 5.45 | 2 |
| A2QKT4 | Probable mannan endo-1,4-beta-mannosidase A OS | [Carbohydrate metabolism](http://www.uniprot.org/keywords/KW-0119) | Glycosidase, Hydrolase | [Secreted](http://www.uniprot.org/keywords/KW-0964) | *Aspergillus niger* | 6.79 | 2 |
| A2QV36 | Probable pectate lyase A OS | Carbohydrate metabolism,Polysaccharide degradation | Lyase | Secreted | *Aspergillus niger* | 29.10 | 6 |
| B0XT32 | Probable pectate lyase A OS | Carbohydrate metabolism,Polysaccharide degradation | Lyase | Secreted | *Aspergillus fumigata* | 16.51 | 4 |
| A2R3I1 | Probable pectin lyase A OS | Carbohydrate metabolism,Polysaccharide degradation | Lyase | Secreted | *Aspergillus niger* | 8.97 | 3 |
| Q8X077 | Probable proteasome subunit alpha type-2 OS | ubiquitin-dependent protein catabolic process | Hydrolase, Protease, Threonine protease | Cytoplasm, Nucleus, Proteasome | *Neurospora crassa* | 13.65 | 2 |
| Q9C2L8 | Probable Ras-related protein Rab7 OS | transport | GTP binding | Cell membrane, Membrane | *Neurospora crassa* | 25.37 | 5 |
| Q9P727 | Probable succinyl-CoA ligase [ADP-forming] subunit alpha, mitochondrial OS | [Tricarboxylic acid cycle](http://www.uniprot.org/keywords/KW-0816) | Ligase | Mitochondrion | *Neurospora crassa* | 7.51 | 2 |
| P87153 | Probable T-complex protein 1 subunit eta OS | [protein folding](http://www.ebi.ac.uk/QuickGO/GTerm?id=GO:0006457) | Chaperone | [Cytoplasm](http://www.uniprot.org/keywords/KW-0963) | *Schizosaccharomyces pombe* | 4.66 | 2 |
| A2QGR5 | Probable Xaa-Pro aminopeptidase P OS |  | Protease |  | *Aspergillus niger* | 8.47 | 4 |
| A2QKF6 | Probable Xaa-Pro aminopeptidase pepP OS |  | Protease |  | *Aspergillus niger* | 21.03 | 7 |
| P38624 | Proteasome subunit beta type-1 OS | proteasomal ubiquitin-independent protein catabolic process | Hydrolase, Protease, Threonine protease | Cytoplasm, Nucleus, Proteasome | *Saccharomyces cerevisiae* | 13.95 | 3 |
| Q12730 | Protein disulfide-isomerase OS | cell redox homeostasis | Isomerase |  | *Aspergillus niger* | 27.38 | 11 |
| Q00216 | Protein disulfide-isomerase tigA OS | [Stress response](http://www.uniprot.org/keywords/KW-0346) | Isomerase | Endoplasmic reticulum | *Aspergillus niger* | 18.94 | 5 |
| Q09173 | Protein phosphatase 2C homolog 3 OS | protein dephosphorylation | Hydrolase, Protein phosphatase | cytosol, nucleus | *Schizosaccharomyces pombe* | 4.11 | 2 |
| A2QBZ0 | Protein transport protein sec31 OS | protein transport |  | Cytoplasmic vesicle, Endoplasmic reticulum, Membrane | *Aspergillus niger* | 1.67 | 2 |
| A2QBH6 | Putative glutathione-dependent formaldehyde-activating enzyme OS | formaldehyde catabolic process | Lyase |  | *Aspergillus niger* | 23.56 | 3 |
| O43099 | Putative peroxiredoxin pmp20 OS | Stress response | Oxidoreductase, Peroxidase | Peroxisome | *Aspergillus fumigata* | 44.64 | 2 |
| Q5ASN8 | Putative peroxiredoxin pmp20 OS | Stress response | Oxidoreductase, Peroxidase | Peroxisome, extracellular region, intracellular | *Aspergillus nidulans* | 20.83 | 3 |
| P0CL19 | Putative ribose 5-phosphate isomerase OS | [carbohydrate metabolic process](http://www.ebi.ac.uk/QuickGO/GTerm?id=GO:0005975) | Isomerase |  | *Coccidioides immitis* | 10.43 | 2 |
| A7GH18 | Pyridoxal biosynthesis lyase PdxS OS | Pyridoxine biosynthesis | Lyase |  | *Clostridium botulinum* | 5.17 | 2 |
| Q9UW83 | Pyridoxine biosynthesis protein pyroA OS | Pyridoxine biosynthesis | catalytic activity | extracellular region | *Aspergillus nidulans* | 9.54 | 2 |
| P42883 | Pyrimidine precursor biosynthesis enzyme THI12 OS | Thiamine biosynthesis |  |  | *Saccharomyces cerevisiae* | 5.00 | 2 |
| Q9HES8 | Pyruvate carboxylase OS | [Gluconeogenesis](http://www.uniprot.org/keywords/KW-0312) | Ligase | Cytoplasm | *Aspergillus niger* | 18.54 | 18 |
| Q12669 | Pyruvate kinase OS | [Glycolysis](http://www.uniprot.org/keywords/KW-0324) | Kinase, Transferase |  | *Aspergillus niger* | 17.49 | 8 |
| P25415 | Quinate dehydrogenase OS | [Quinate metabolism](http://www.uniprot.org/keywords/KW-0672) | [Oxidoreductase](http://www.uniprot.org/keywords/KW-0560) |  | *Aspergillus nidulans* | 7.90 | 2 |
| Q8NJK5 | Rhamnogalacturonate lyase A (Fragment) OS | Carbohydrate metabolism,Polysaccharide degradation | Lyase | Secreted | *Aspergillus niger* | 12.63 | 4 |
| P19791 | Ribonuclease M OS | RNA binding | Endonuclease, Hydrolase, Nuclease |  | *Aspergillus saitoi* PE | 18.07 | 3 |
| A3LP13 | Ribose-5-phosphate isomerase OS | [pentose-phosphate shunt, non-oxidative branch](http://www.ebi.ac.uk/QuickGO/GTerm?id=GO:0009052) | Isomerase | Cytoplasm | *Scheffersomyces stipitis* | 10.97 | 2 |
| B8HQS5 | Ribulose bisphosphate carboxylase large chain OS | photorespiration | Lyase, Monooxygenase, Oxidoreductase |  | *Cyanothece* sp. | 8.82 | 3 |
| Q870G1 | Saccharopine dehydrogenase [NAD(+), L-lysine-forming] OS | [Amino-acid biosynthesis](http://www.uniprot.org/keywords/KW-0028) | Oxidoreductase |  | *Aspergillus nidulans* | 6.93 | 3 |
| P48466 | S-adenosylmethionine synthase OS | [One-carbon metabolism](http://www.uniprot.org/keywords/KW-0554) | Transferase | Cytoplasm | *Neurospora crassa* | 10.13 | 2 |
| A4J945 | S-adenosylmethionine synthase OS | [One-carbon metabolism](http://www.uniprot.org/keywords/KW-0554) | Transferase | Cytoplasm | *Desulfotomaculum reducens* | 8.82 | 2 |
| A6Q1Z8 | S-adenosylmethionine synthase OS | [One-carbon metabolism](http://www.uniprot.org/keywords/KW-0554) | Transferase | Cytoplasm | *Nitratiruptor sp.* | 6.22 | 2 |
| P37291 | Serine hydroxymethyltransferase, cytosolic OS | [One-carbon metabolism](http://www.uniprot.org/keywords/KW-0554) | Transferase | Cytoplasm | *Saccharomyces cerevisiae* | 3.84 | 2 |
| O14018 | Serine--tRNA ligase, cytoplasmic OS | [Protein biosynthesis](http://www.uniprot.org/keywords/KW-0648) | Aminoacyl-tRNA synthetase, Ligase | Cytoplasm | *Schizosaccharomyces pombe* | 9.33 | 3 |
| P52718 | Serine-type carboxypeptidase F OS |  | Carboxypeptidase, Hydrolase, Protease |  | *Aspergillus niger* | 3.77 | 2 |
| A1CRG9 | Small COPII coat GTPase sar1 OS | [Transport](http://www.uniprot.org/keywords/KW-0813) | Hydrolase | [Membrane](http://www.uniprot.org/keywords/KW-0472) | *Aspergillus clavatus* | 11.64 | 2 |
| C0NRX4 | S-methyl-5'-thioadenosine phosphorylase OS | [Purine salvage](http://www.uniprot.org/keywords/KW-0660) | Glycosyltransferase, Transferase |  | *Ajellomyces capsulata* | 7.99 | 2 |
| Q4WWS3 | Sorting nexin-3 OS | Transport | phosphatidylinositol binding | Cytoplasm, Golgi apparatus, Membrane | *Aspergillus fumigata* | 18.31 | 3 |
| Q12230 | Sphingolipid long chain base-responsive protein LSP1 OS | response to heat, endocytosis | lipid binding | Cytoplasm | *Saccharomyces cerevisiae* | 7.92 | 2 |
| P33295 | Subtilisin-like serine protease pepC OS |  | Hydrolase, Protease, Serine protease |  | *Aspergillus niger* | 18.39 | 7 |
| P47052 | Succinate dehydrogenase [ubiquinone] flavoprotein subunit 2, mitochondrial OS | Electron transport, Transport, Tricarboxylic acid cycle | Oxidoreductase | Mitochondrion inner membrane | *Saccharomyces cerevisiae* | 3.79 | 2 |
| Q00711 | Succinate dehydrogenase [ubiquinone] flavoprotein subunit, mitochondrial OS | Electron transport, Transport, Tricarboxylic acid cycle | Oxidoreductase | Mitochondrion inner membrane | *Saccharomyces cerevisiae* | 3.44 | 2 |
| P0AGF1 | Succinyl-CoA ligase [ADP-forming] subunit alpha OS | [Tricarboxylic acid cycle](http://www.uniprot.org/keywords/KW-0816) | Ligase |  | *Escherichia coli* O157:H7 | 15.22 | 3 |
| Q51567 | Succinyl-CoA ligase [ADP-forming] subunit alpha OS | [Tricarboxylic acid cycle](http://www.uniprot.org/keywords/KW-0816) | Ligase |  | *Pseudomonas aeruginosa* | 15.25 | 3 |
| A4W879 | Succinyl-CoA ligase [ADP-forming] subunit beta OS | [Tricarboxylic acid cycle](http://www.uniprot.org/keywords/KW-0816) | Ligase |  | *Enterobacter* sp. | 6.70 | 2 |
| Q8NJN1 | Sulfate adenylyltransferase OS | [Amino-acid biosynthesis](http://www.uniprot.org/keywords/KW-0028) | Nucleotidyltransferase, Transferase | Cytoplasm | *Aspergillus niger* | 18.64 | 9 |
| A2QMY6 | Superoxide dismutase [Cu-Zn] OS | [superoxide metabolic process](http://www.ebi.ac.uk/QuickGO/GTerm?id=GO:0006801) | Antioxidant, Oxidoreductase | Cytoplasm | *Aspergillus niger* | 44.81 | 5 |
| P00448 | Superoxide dismutase [Mn] OS | [response to oxidative stress](http://www.ebi.ac.uk/QuickGO/GTerm?id=GO:0006979) | Oxidoreductase | Cytoplasm | *Escherichia coli* | 28.16 | 3 |
| Q3MSU9 | Superoxide dismutase [Mn], mitochondrial OS | [superoxide metabolic process](http://www.ebi.ac.uk/QuickGO/GTerm?id=GO:0006801) | Oxidoreductase | [Mitochondrion](http://www.uniprot.org/keywords/KW-0496) | *Aspergillus niger* | 19.52 | 4 |
| Q9UUZ9 | Thiamine thiazole synthase OS | Thiamine biosynthesis,response to stress | [1. metal ion binding](http://www.ebi.ac.uk/QuickGO/GTerm?id=GO:0046872) | Cytoplasm, Nucleus | *Aspergillus oryzae* | 8.26 | 2 |
| O42700 | Transaldolase OS | [Pentose shunt](http://www.uniprot.org/keywords/KW-0570) | Transferase | cytoplasm | *Schizosaccharomyces pombe* | 12.11 | 2 |
| B3Q0T5 | Transaldolase OS | [pentose-phosphate shunt, non-oxidative branch](http://www.ebi.ac.uk/QuickGO/GTerm?id=GO:0009052) | Transferase | cytoplasm | *Rhizobium etli* | 6.54 | 2 |
| Q9HGY8 | Triosephosphate isomerase OS | Gluconeogenesis, Glycolysis, Pentose shunt | Isomerase |  | *Aspergillus oryzae* | 19.52 | 4 |
| P13228 | Tryptophan synthase OS | [Amino-acid biosynthesis](http://www.uniprot.org/keywords/KW-0028) | Lyase |  | *Neurospora crassa* | 3.25 | 2 |
| P24633 | Tubulin alpha-1 chain OS | Microtubule polymerization, nuclear division | structural constituent of cytoskeleton | Cytoplasm, Cytoskeleton, Microtubule | *Aspergillus nidulans* | 9.35 | 4 |
| P24634 | Tubulin alpha-2 chain OS | Mmicrotubule polymerization, nuclear division | structural constituent of cytoskeleton | Cytoplasm, Cytoskeleton, Microtubule | *Aspergillus nidulans* | 8.43 | 3 |
| P22012 | Tubulin beta chain OS | Microtubule polymerization, nuclear division | structural constituent of cytoskeleton | Cytoplasm, Cytoskeleton, Microtubule | *Aspergillus flavus* PE | 17.63 | 6 |
| P19848 | Ubiquitin OS | [protein ubiquitination](http://www.ebi.ac.uk/QuickGO/GTerm?id=GO:0016567) |  | Cytoplasm, Nucleus | *Coprinus congregatus* PE | 35.53 | 3 |
| P22515 | Ubiquitin-activating enzyme E1 1 OS | [protein ubiquitination](http://www.ebi.ac.uk/QuickGO/GTerm?id=GO:0016567) | Ligase | Cytoplasm, Nucleus | *Saccharomyces cerevisiae* | 2.73 | 2 |
| P52495 | Ubiquitin-activating enzyme E1 1 OS | protein ubiquitination | Ligase | Cytoplasm, Nucleus | *Candida albicans* | 2.15 | 2 |
| Q00511 | Uricase OS | [Purine metabolism](http://www.uniprot.org/keywords/KW-0659) | [Oxidoreductase](http://www.uniprot.org/keywords/KW-0560) | Peroxisome | *Aspergillus flavus* | 6.95 | 2 |
| O42630 | Vacuolar protease A OS | [Virulence](http://www.uniprot.org/keywords/KW-0843) | Aspartyl protease, Hydrolase, Protease | Secreted, Vacuole | *Aspergillus fumigata* | 6.28 | 2 |
| P11592 | V-type proton ATPase catalytic subunit A OS | transport | Hydrolase | fungal-type vacuole membrane | *Neurospora crassa* | 6.26 | 2 |
| Q9UVJ8 | V-type proton ATPase catalytic subunit A OS | transport | Hydrolase | fungal-type vacuole membrane | *Ashbya gossypii* | 3.89 | 2 |
| P22550 | V-type proton ATPase subunit B OS | transport | Hydrolase | proton-transporting V-type ATPase, V1 domain | *Candida tropicalis* | 6.07 | 4 |
| Q9P8K9 | Woronin body major protein OS | cellular response to farnesol | translation elongation factor activity | [Peroxisome](http://www.uniprot.org/keywords/KW-0576) | *Aspergillus nidulans* | 7.24 | 2 |

Table S2 List of the secreted proteins in SSF of Pu-erh tea

| Accession | Description | Biological process | Function | Organism |
| --- | --- | --- | --- | --- |
| Q8TFN0 | Nucleoside diphosphate kinase OS | Nucleotide metabolism |  | *Aspergillus nidulans* |
| Q03134 | Probable formate dehydrogenase OS | Acetate metabolic process |  | *A. nidulans* |
| Q9UW83 | Pyridoxine biosynthesis protein pyroA OS | Pyridoxine biosynthesis |  | *A. nidulans* |
| B8N106 | Alkaline protease 1 OS | Proteolysis | Assimilation of proteinaceous substrates | *A. flavus* |
| A2R3L3 | Aspartic protease pep1 OS | Proteolysis | Assimilation of proteinaceous substrates | *A. niger* |
| P79021 | Probable alpha-L-arabinofuranosidase axhA OS | Carbohydrate metabolism, Polysaccharide degradation | Hydrolysis of xylan | *A. tubingensis* |
| A2Q7I0 | Probable endo-1,4-beta-xylanase B OS | Carbohydrate metabolism, Polysaccharide degradation | Hydrolysis of xylan | *A. niger* |
| A2QFV7 | Probable endo-1,4-beta-xylanase C OS | Carbohydrate metabolism, Polysaccharide degradation | Hydrolysis of xylan | *A. niger* |
| A2QA27 | Probable exo-1,4-beta-xylosidase xlnD OS | Carbohydrate metabolism, Polysaccharide degradation | Hydrolysis of xylan | *A. niger* |
| O42814 | Alpha-glucuronidase A OS | Carbohydrate metabolism, Polysaccharide degradation, Xylan degradation | Hydrolysis of xylan | *A. tubingensis* |
| Q8NK90 | Alpha-N-arabinofuranosidase A OS | Carbohydrate metabolism, Polysaccharide degradation | Degradation of arabinoxylan | *A. kawachii* |
| P42255 | Alpha-N-arabinofuranosidase B OS | Carbohydrate metabolism, Polysaccharide degradation | Hydrolyze 1,5-, 1,3- and 1,2-alpha-linkages in L-arabinofuranosyl oligosaccharides, and polysac-charides containing terminal non-reducing L-arabinofuranoses in side chains | *A. niger* |
| Q8NK89 | Alpha-N-arabinofuranosidase B OS | Carbohydrate metabolism, Polysaccharide degradation |  | *A. kawachii* |
| P17872 | Pectinesterase OS | Cell wall biogenesis/degradation | Maceration and soft-rotting of plant tissue | *A. tubingensis* |
| A2R2S6 | Probable alpha-galactosidase D OS | Carbohydrate metabolism, Polysaccharide degradation | Hydrolyzes of alpha-D-galactoside, oligosaccharides and polysaccharides | *A. niger* |
| Q0CVX4 | Probable alpha-galactosidase D OS | Carbohydrate metabolism,Polysaccharide degradation |  | *A. terreus* |
| Q9Y7F8 | Probable arabinogalactan endo-1,4-beta-galactosidase A OS | Carbohydrate metabolism,Polysaccharide degradation |  | *A. tubingensis* |
| A2QAN3 | Probable beta-galactosidase A OS | Carbohydrate metabolism,Polysaccharide degradation | Cleaves beta-linked terminal galactosyl residues from gangliosides, glycoproteins, and glycosaminoglycans | *A. niger* |
| A2QA64 | Probable beta-galactosidase B OS | Carbohydrate metabolism,Polysaccharide degradation | Cleaves beta-linked terminal galactosyl residues from gangliosides, glycoproteins, and glycosaminoglycans | *A. niger* |
| A2QL84 | Probable beta-galactosidase C OS | Carbohydrate metabolism,Polysaccharide degradation | Cleaves beta-linked terminal galactosyl residues from gangliosides, glycoproteins, and glycosaminoglycans | *A. niger* |
| A2RBC2 | Probable carboxypeptidase An18g06210 OS | proteolysis |  | *A. niger* |
| A2R8F8 | Probable endopolygalacturonase A OS | Cell wall biogenesis/degradation | Maceration and soft-rotting of plant tissue. Hydrolyzes the 1,4-alpha glycosidic bonds of de-esterified pectate | *A. niger* |
| A2QHG0 | Probable exopolygalacturonase B OS | Cell wall biogenesis/degradation | Hydrolyzing the terminal glycosidic bond of polygalacturonic acid and oligogalacturonates | *A. niger* |
| A2RAR6 | Probable glucan 1,3-beta-glucosidase A OS | Carbohydrate metabolism,Polysaccharide degradation | Metabolism of beta-glucan | *A. niger* |
| A2QH21 | Probable glucan endo-1,3-beta-glucosidase eglC OS | Carbohydrate metabolism,Polysaccharide degradation | Degradation of beta-glucan | *A. niger* |
| A2QKT4 | Probable mannan endo-1,4-beta-mannosidase A OS | Carbohydrate metabolism | Depolymerization of galactomannans | *A. niger* |
| A2QV36 | Probable pectate lyase A OS | Carbohydrate metabolism,Polysaccharide degradation | Depolymerization of pectin | *A. niger* |
| B0XT32 | Probable pectate lyase A OS | Carbohydrate metabolism,Polysaccharide degradation | Depolymerization of pectin | *A. fumigata* |
| A2R3I1 | Probable pectin lyase A OS | Carbohydrate metabolism,Polysaccharide degradation | Depolymerization of pectin | *A. niger* |
| Q8NJK5 | Rhamnogalacturonate lyase A (Fragment) OS | Carbohydrate metabolism,Polysaccharide degradation | Degrades the rhamnogalacturonan I (RG-I) backbone of pectin | *A. niger* |
| A2QK83 | Probable endo-xylogalacturonan hydrolase A OS | Carbohydrate metabolism,Polysaccharide degradation | Degradation of xylogalacturonan which is with xylose, and one important component of the hairy regions of pectin | *A. niger* |
| A5AAG2 | Probable arabinan endo-1,5-alpha-L-arabinosidase C OS | Carbohydrate metabolism,Polysaccharide degradation | Degradation of pectin | *A. niger* |
| A2RB93 | Probable arabinogalactan endo-1,4-beta-galactosidase A OS | Carbohydrate metabolism,Polysaccharide degradation | Degradation of plant cell wall polysaccharides, and more particularly of hairy regions of pectin | *A. niger* |
| O42630 | Vacuolar protease A OS | Virulence |  | *A. fumigata* |
| A2R989 | Probable beta-glucosidase I OS | Carbohydrate metabolism,Polysaccharide degradation | Degradation of cellulosic biomass | *A. niger* |
| A5ABF5 | Probable beta-glucosidase M OS | Carbohydrate metabolism,Polysaccharide degradation | Degradation of cellulosic biomass | *A. niger* |
| P87076 | Beta-glucosidase A OS | Carbohydrate metabolism, Cellulose degradation, Polysaccharide degradation | Degradation of cellulosic biomass | *A. kawachii* |
| Q96WQ8 | Probable endo-beta-1,4-glucanase B OS | Carbohydrate metabolism, Cellulose degradation, Polysaccharide degradation | Degradation of complex natural cellulosic substrates | *A. kawachii* |
| A2R0Z6 | Probable feruloyl esterase B OS | Carbohydrate metabolism,Polysaccharide degradation | Degradation of plant cell walls | *A. niger* |
| Q5B2Q4 | Probable 1,4-beta-D-glucan cellobiohydrolase A OS | Carbohydrate metabolism,Cellulose degradation,Polysaccharide degradation, | Conversion of cellulose to glucose | *A. nidulans* |
| Q877A8 | Catalase B OS | [Hydrogen peroxide](http://www.uniprot.org/keywords/KW-0376) | protect cells from the toxic effects of hydrogen peroxide | *Aspergillus oryzae* |
| A2Q7T1 | Catalase-peroxidase OS | Hydrogen peroxide | Bifunctional enzyme with both catalase and broad-spectrum peroxidase activity | *Aspergillus niger* |
| Q5ASN8 | Putative peroxiredoxin pmp20 OS | Stress response | Involved in osmoadaptation | *Aspergillus nidulans* |

Figure S1. Rarefaction curves indicating the observed number of operational taxonomic units (OTUs) at a genetic distance of 3% of bacteria (a) and fungi (b) in fermented tea leaves.

Figure S2. Amounts of proteins extracted at various times

^a^ Indicates there is no significant difference.

Figure S3. LC-MS/MS chromatograms of the trypsin hydrolyzed peptides of proteins (b) extracted from fermented tea leaves collected on day 21.

Figure S4. Comparison of the bacterial community structure based on metagenomic (a) and metaproteomic (b) analyses at the phylum level.

Figure S5. Comparison of fungal community structure based on metagenomic (a) and metaproteomic (b) analyses at the genus level.
